# Murine model of high bone mass osteogenesis imperfecta exhibits bone matrix hyper-mineralization, misaligned mineral crystals, and altered osteoblast differentiation

**DOI:** 10.1038/s41413-026-00531-7

**Published:** 2026-06-15

**Authors:** Aileen M. Barnes, M. Helen Rajpar, Joseph E. Perosky, Stéphane Blouin, Basma Khoury, MaryAnn Weis, Theresa Hefferan, Alberta Derkyi, Gali Guterman-Ram, Ghazal Hedjazi, Kiersten Campbell, Chris Stephan, David R. Eyre, Ryan K. Dale, Peter Fratzl, Kenneth M. Kozloff, Nadja Fratzl-Zelman, Joan C. Marini

**Affiliations:** 1 Section on Heritable Disorders of Bone and Extracellular Matrix, Eunice Kennedy Shriver National Institute of Child Health and Human Development, National Institutes of Health, Bethesda, MD USA; 2 Department of Orthopaedic Surgery, University of Michigan, Ann Arbor, MI USA; 3 Ludwig Boltzmann Institute of Osteology at Hanusch Hospital of OEGK and AUVA Trauma Centre Meidling, 1st Med. Department Hanusch Hospital, Vienna, Austria; 4 Vienna Bone and Growth Center, Vienna, Austria; 5 Department of Orthopaedics and Sports Medicine, University of Washington, Seattle, WA USA; 6 Biomaterials and Histomorphometry Core Laboratory, Mayo Clinic, Rochester, MN USA; 7 Office of the Clinical Director, Eunice Kennedy Shriver National Institute of Child Health and Human Development, National Institutes of Health, Bethesda, MD USA; 8 Bioinformatics & Scientific Programming Core, Eunice Kennedy Shriver National Institute of Child Health and Human Development, NIH, Bethesda, MD USA; 9 Max Planck Institute of Colloids and Interfaces, Potsdam, Germany; 10 Present Address: Faculty of Biomedical Engineering, Technion-Israel Institute of Technology, Haifa, Israel

**Keywords:** Bone quality and biomechanics, Calcium and phosphate metabolic disorders

## Abstract

Osteogenesis imperfecta (OI), characterized by bone fragility and low bone mass, is predominantly caused by mutations in type I collagen. High bone mass OI (HBM OI) is a rare form caused by heterozygous missense mutations at the type I procollagen C-propeptide cleavage site. Knock-in HBM OI mice were generated to elucidate the effect of this mutation on cells and bone. HBM OI murine femora contain increased monomeric pro-α1(I)C-propeptide and pC-collagen; their bone collagen fibrils have a “barbed-wire” appearance. Decreased C-propeptide cleavage diminishes bone strength. HBM OI femora are extremely brittle, with thin cortices, decreased BV/TV, and fracture load. The cortical bone has increased mineral content, with thinner, more disorganized mineral particles. Increased expression of ossification genes in both murine and human HBM OI osteoblasts during in vitro differentiation and increased mineral deposition in culture indicate impaired C-propeptide processing affects cellular processes related to mineralization, rather than being a passive matrix process. Gene ontology analysis of RNA-seq data from differentiating HBM OI osteoblasts revealed top upregulated pathways for ossification, mineralization, and osteoblast differentiation (5–25×) while top-downregulated pathways involved cellular adhesion, migration, and angiogenesis (5–10×), all related to cell-matrix interactions. Moreover, the HBM matrix affects osteoblast function. WT osteoblasts plated on HBM OI decellularized matrix in vitro showed less punctate vinculin, increased peripheral actin staining, and the presence of lamellipodia, suggesting a decrease in cellular adhesion. Insights into the mechanism of HBM OI mineralization may lead to improved therapies for HBM OI and low bone mass conditions.

## Introduction

Type I collagen, a fibril-forming collagen, is the major structural protein in bone, skin, and tendon. In bone tissues, collagen fibrils provide plasticity in addition to serving as a scaffold for hydroxyapatite crystals, which endow bone with stiffness and the ability to withstand stress.^1^ The C-propeptides of procollagens are highly conserved domains important for chain alignment and association.^2–4^ When procollagen chains have been aligned at the C-propeptide domain, folding proceeds from the C- to N-terminus in a zipper-like fashion.^5^ Once folded within the osteoblast, procollagen is secreted, and the propeptides are cleaved in the pericellular space by specific proteases, releasing the mature fibril. The type I collagen C-propeptide is specifically cleaved at an alanine-aspartic acid bond in the C-telopeptide by bone morphogenetic protein 1 (BMP-1), assisted by procollagen C-proteinase enhancer 1 (PCPE1).^6–8^ Moreover, early biochemical studies reported that cleaved trimeric C-propeptides had a role in cell attachment, endothelial migration, and in the downregulation of collagen synthesis.^9–12^

Classical osteogenesis imperfecta (OI), or brittle bone disease, is caused by structural or quantitative defects in either of the type I collagen genes, *COL1A1* or *COL1A2*.^13^ Common features of OI include severe bone fragility, low bone mass, deformity of long bones, and short stature. While most collagen structural defects consist of glycine substitutions or splicing defects within the helical region,^13^ changes affecting the collagen propeptides lead to distinct phenotypes. Collagen mutations that eliminate the N-propeptide cleavage site (usually by exon skipping) cause the arthrochalasia type of Ehlers-Danlos Syndrome (EDS), which is characterized by joint hypermobility and congenital bilateral hip dislocation.^14^ Additionally, mutations located in the first 90 residues of the collagen helix led to a distinctive, moderately severe, OI-EDS phenotype that combines a collagen structural defect with diminished N-propeptide processing.^15^ In contrast, substitutions of conserved residues in the C-propeptide cause mild to lethal classical OI due to endoplasmic reticulum (ER) mislocalization of collagen, delayed chain incorporation, and partial impairment of C-propeptide processing.^16–18^

In 2011, we identified mutations in the alanine-aspartic acid C-propeptide cleavage site of type I collagen in two OI patients (COL1A1 p.Asp1219Asn; COL1A2 p.Ala1118Thr). These patients had a phenotype of fractures, normal stature, increased or normal bone density, and high bone mineralization, which we designated high bone mass (HBM) OI^19^. Currently, at least ten patients have been identified with cleavage site mutations, encompassing all four cleavage site residues on the two type I procollagen chains.^19–27^ Bone mineral density (BMD) measurements reported for patients with defects in the C-propeptide cleavage site yield increased or normal DXA (dual x-ray absorptiometry) z-scores. At the tissue level, HBM OI patients exhibit bone areas with abnormally high and abnormally low matrix mineralization, as measured by quantitative backscattered electron imaging (qBEI),^19^ and can have variations in areal bone density throughout their skeleton on whole body DXA.^25^

In this study, we investigated the role of the C-propeptide in bone mass accrual and mineralization by generating a heterozygous HBM OI mouse model. In humans, a single substitution in the C-propeptide cleavage site disrupts C-propeptide cleavage^19^ and leads to an HBM OI phenotype. However, to avoid potential C-propeptide cleavage redundancy in the mouse, both the alanine and aspartic acid residues of the α1(I) procollagen C-propeptide cleavage site were substituted in the same allele with threonine and asparagine, respectively. The amino acid changes reflected the same variants seen in our original HBM OI patients to model the human disease.^19^ The HBM OI mouse reproduces the high tissue mineral density and increased tissue-level mineralization seen in patients, leading to brittleness. Although the collagen helix is structurally normal, the lack of C-propeptide cleavage leads to decreased collagen deposition in the matrix and, paradoxically, abundant unmineralized matrix (osteoid) deposition on bone surfaces. Murine studies reveal that, together with the altered collagen matrix, upregulation of osteoblast differentiation and extracellular matrix genes contribute to the HBM OI phenotype.

## Results

### Generation of the high bone mass osteogenesis imperfecta (HBM OI) murine model

**Fig. 1:**
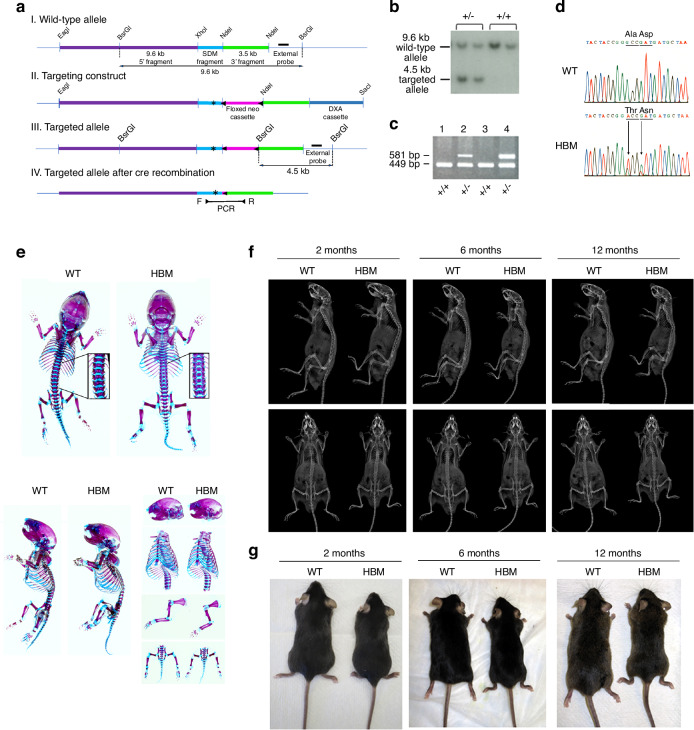
Generation of the high bone mass (HBM) OI mouse model. **a** Targeting constructs for the generation of the HBM OI mouse before and after EIIa-Cre recombination. I. The WT allele, showing restriction enzyme site locations, the 9.6 kb 5′ upstream homology arm, the site-directed mutagenesis (SDM) fragment, and the 3.5 kb 3′ downstream homology arm. II. The targeting construct showing the location of the neo cassette and diphtheria toxin negative selection element. III. The targeted allele showing the fragments used in Southern blotting (4.5 kb fragment, black arrows). IV. The targeted allele after recombination, carrying the knock-in mutation and loss of the neo selection cassette. The PCR primer locations are shown by black triangles (Geno-F, Geno-R) on the knock-in allele. **b** Southern blot of *BsrGI*-digested genomic DNA showing the expected fragment sizes of the WT (9.6 kb) and recombined targeted allele (4.5 kb). **c** Genotyping of HBM OI mouse tail DNA using Geno-F and Geno-R primers produces fragments of 449 bp (WT) and 581 bp (HBM OI). **d** Sequence tracings from osteoblast gDNA show the two heterozygous mutations introduced to the COL1A1 C-propeptide cleavage site to prevent C-propeptide cleavage. **e** Newborn skeletons stained with alizarin red (bone) and alcian blue (cartilage) are grossly normal. HBM OI mice have slightly flared ribs and a minor delay in vertebral mineralization. **f** Radiographs from 2-, 6-, and 12-month-old male mice show that HBM OI mice have osteopenic bones, kyphosis, a narrow thorax, and a flared pelvis. **g** HBM OI mice are visually smaller and thinner than WT littermates at all ages

To investigate the mechanism of HBM OI, heterozygous HBM OI mice were generated by homologous recombination, in which both residues of the α1(I) procollagen C-propeptide cleavage site were substituted (p.Ala1207_Asp1208delinsThrAsn) to block processing of mutant proα1(I) chains. (Fig. 1a). Mutant HBM OI mice were crossed twice with EIIA-Cre mice to excise the floxed neo cassette. Successful recombination of the HBM OI allele was verified by Southern blot of *BsrGI*-digested genomic DNA (Fig. 1b), genotyping (Fig. 1c), and osteoblast gDNA sequencing (Fig. 1d). HBM OI is dominantly inherited; thus, HBM OI mice are heterozygous for the mutant allele.

**Fig. 2:**
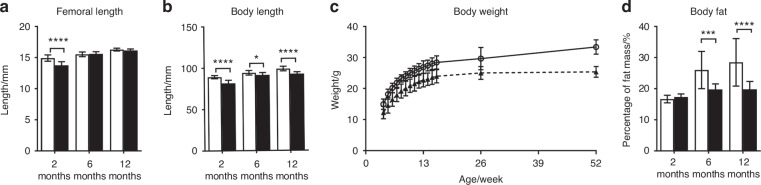
Murine HBM OI physical features. **a** HBM OI femur length is shorter at 2 months of age but normalizes by 6 months. **b** HBM OI mice are shorter in length throughout life. **c** HBM OI mice (filled triangles) weigh significantly less than WT littermates (open circles) throughout life (*P* < 0.001 at all timepoints). **d** The total fat mass percentage, as measured by DXA, is significantly decreased at 6- and 12-months in HBM OI mice (*n* = 9-16 male mice, per age, per genotype). (WT = white bars, HBM OI = black bars). (*P < 0.05, **P < 0.01, ***P < 0.005, ****P < 0.001)

Newborn HBM OI murine skeletons were grossly normal (Fig. 1e), though some had flared rib cages and/or displayed a slight vertebral mineralization delay (see inset). Kyphosis, flared pelvises, where pelvic rami extend more laterally than normal, and contracted ankle joints were increased in HBM OI adults compared to WT littermates (Fig. 1f). HBM OI mice were visibly smaller in size (Fig. 1g) with body length modestly decreased by 3%–8% throughout life (Fig. 2b). Femora were shorter at 2 months, normalizing at 6 months (Fig. 2a). Body weight was 15%–20% lower than WT littermates starting at 4 weeks of age (Fig. 2c). Furthermore, total percent body fat was decreased in 6-, 12-month and 2-y-old mice (Fig. 2d, Fig. S1A).

As HBM OI mice aged, ankle contractions were a uniform finding, and patellar tendons often appeared damaged/thickened. Knee sections showed minimal changes in articular cartilage, without degeneration or signs of osteoarthritis (Fig. S1B). However, radiodense material at the knee joint increased as mice aged (Fig. S1C). Six-month hind limbs revealed heterotopic ossification of the patellar tendon (Fig. S1D) and potential ossification of the Achilles tendon.

We attempted to generate homozygous HBM OI/HBM OI mice, in which all proα(I)I chains were uncleavable. All HBM OI dams developed dystocia and could not give birth. μCT scans of the pelvis of breeding-aged HBM OI females revealed a narrower birth canal with apparent excess calcification (Fig. S1E). Pups were retrieved by Cesarean sections and fostered. Many homozygous pups had abdominal hernias and died perinatally. Only two homozygous HBM OI/HBM OI mice survived beyond the perinatal period; one lived 10 days (Fig. S2A) and another 5 weeks (Fig. S2B, C). Radiographs revealed that homozygotes had strikingly decreased size compared to littermates, and multiple fractures of ribs and lower limbs (Fig. S2A–C). The 5-week pup weighed ~20% of its littermates (3.2 g vs. 15.2 g ± 1.8 g).

### HBM OI murine osteoblasts and matrix have impaired procollagen C-propeptide cleavage

**Fig. 3:**
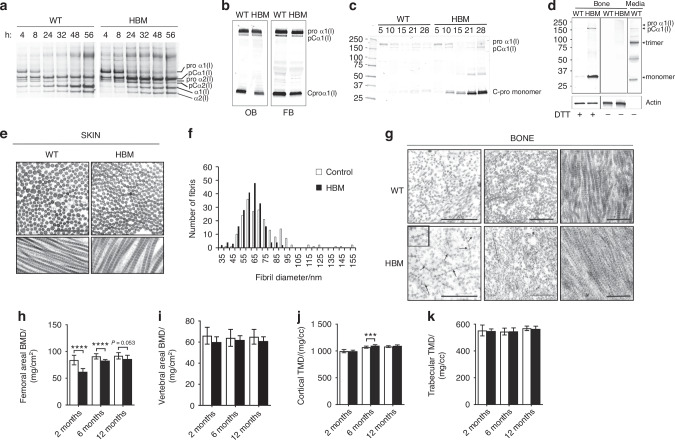
pC-α1(I) collagen and C-propeptide are incorporated into bone tissue. **a** Pericellular processing of ^3^H-labeled type I procollagen in osteoblast conditioned media is decreased in HBM OI vs WT, with an increase in pC-α1(I) and a decrease in mature α1(I) collagen chains (*n* = 2). **b** Representative COL1A1 C-propeptide western blot of conditioned medium from osteoblasts and fibroblasts (*n* = 5 WT/HBM paired samples, per cell type). There is an approximately 25%–30% decrease in collagen processing. **c** COL1A1 C-propeptide western blot of extracellular matrix harvested from differentiated osteoblasts shows an increase of C-propeptide present in the matrix over the differentiation time course (5–28 days in osteogenic media) (*n* = 2–3). **d** COL1A1 C-propeptide western blot of long bone tissue (femora and tibiae) lysate from 2-month-old mice reveals that HBM OI bone incorporates pC-α1(I) and monomeric C-propeptide into bone tissue. The trimeric C-propeptide is not incorporated into bone (*n* = 2). **e** Transmission electron micrographs of dermal fibrils of 2-month-old mice show a normal D-period banding pattern (bottom panels), with significantly decreased fibril diameter and variability, lacking large fibrils (top panels). Scale bar = 500 nm, *n* = 200 fibrils measured for each genotype, measured from 3–4 separate images. **f** Histogram of WT and HBM OI fibril measurements (*n* = 200). **g** Transmission electron micrographs of bone collagen fibrils of 2-month-old WT and HBM OI mice. The middle and left panels show smaller bone fibril diameters and some fibrils with a “barbed-wire” appearance (black arrows, inset) in HBM OI. The longitudinal sections (right panels) display thinner fibrils in HBM OI, with less distinct D-period banding. Scale bars = 500 nm. **h**, **i** Femoral and vertebral areal bone density (aBMD) were measured at 2-, 6-, and 12-months of age (*n* = 10, each genotype). Femoral aBMD is decreased in HBM OI vs WT at 2- and 6-months of age but approaches WT values by 12 months (**h**). Vertebral aBMD is normal in HBM OI mice (**i**). **j**, **k** HBM OI cortical and trabecular tissue mineral density (TMD) measured by µCT shows a similar volumetric bone density to WT at all ages. (WT = white bars, HBM OI = black bars). (***P < 0.005, ****P < 0.001)

To confirm decreased C-propeptide cleavage in HBM OI mice, collagen processing was verified using cultured HBM OI calvarial osteoblasts. Gel electrophoresis of ^3^H-labeled collagen bands showed an increase in pC-α1(I) collagen (procollagen with uncleaved C-propeptide) and a decrease in mature α1(I) and α2(I) collagen chains in cell culture media over time (Fig. 3a). Further, in western blot, the cleavage of the C-propeptide was decreased in both FB [(75.3 ± 18.3)%] and OB [(66.7 ± 21.6)%] compared to WT (Fig. 3b). Taken together, these data indicate the presence of diminished collagen processing in these mice. In addition, increased levels of pC-α1(I) collagen and α1(I) C-propeptide monomer were present in mature HBM OI osteoblast matrix, as well as in 2-month (Fig. 3c, d) and 1-year-old HBM OI femur extracts (Fig. S3A). Despite the abundance of C-propeptide monomer in the bone, trimeric C-propeptide was not present in WT or HBM OI femur extracts (Fig. 3d).

Incorporation of uncleaved pC-α1(I) into dermal collagen fibrils caused a significant decrease in fibril diameter (WT = 69.1 ± 18.4 nm; HBM = 60.2 ± 9.6 nm, *P* < 0.000 1, Fig. 3e, f). HBM OI bone fibrils are also smaller in diameter than WT and have a “barbed-wire” appearance (Fig. 3g, black arrows, inset), indicating that the C-propeptide remains uncleaved when incorporated into fibrils. Fibrils from the homozygous P10 pup showed a similar pattern, with a greater decrease in fibril diameters vs. WT skin (WT = 49.2 ± 4.7 nm; HBM = 35.5 ± 3.4 nm, *P* < 0.000 1) and bone tissues, and with irregular cross-sectional margins and irregular widths (Fig. S2D, E).

### HBM OI bones are thinner and extremely brittle, but have normal bone density

HBM OI bone was examined by µCT and biomechanics. Cortical thickness and area were both reduced, and marrow area correspondingly increased with age (Table 1, Fig. S3B), features seen to a greater extent in the 5-week-old homozygote (Fig. S2F). Trabecular HBM OI bone volume (BV/TV) was consistently lower than WT (Table 1) due to a significant decrease in trabecular number, not thickness.

**Table 1:** µCT parameters of HBM-OI cortical and trabecular bone

Cortical	2 months WT	2 months HBM-OI	*P*-value	6 months WT	6 months HBM-OI	*P*-value	12 months WT	12 months HBM-OI	*P*-value
BMC/mg	1.76 ± 0.47	1.37 ± 0.25	**0.021**	2.19 ± 0.19	1.86 ± 0.21	**0.000 3**	2.66 ± 0.27	2.31 ± 0.10	**0.002**
BMD/(mg/cc)	803.05 ± 97.2	786.44 ± 133.14	0.734	1 071.00 ± 19.85	1 098.29 ± 19.44	**0.002**	853.69 ± 74.88	803.30 ± 13.22	0.062
TMC/mg	1.56 ± 0.36	1.20 ± 0.19	**0.007**	2.19 ± 0.19	1.86 ± 0.20	**0.000 3**	2.37 ± 0.22	2.01 ± 0.10	**0.000 2**
TMD/(mg/cc)	996.68 ± 30.60	995.82 ± 18.92	0.937	1 071.38 ± 19.81	1 098.45 ± 19.21	**0.002**	1 082.62 ± 15.96	1 096.24 ± 17.44	0.078
Mean thickness/mm	0.17 ± 0.02	0.14 ± 0.01	**0.000 2**	0.21 ± 0.01	0.17 ± 0.01	**2.17E-11**	0.21 ± 0.01	0.17 ± 0.01	**1.18E-08**
Bending moment/mm^4^	0.12 ±0.03	0.10 ±0.03	0.204	0.15 ± 0.02	0.17 ± 0.04	0.336	0.17 ± 0.02	0.16 ± 0.02	0.367
Inner Perimeter/mm	4.05 ± 0.23	4.03 ± 0.29	0.893	3.93 ± 0.32	4.06 ± 0.32	0.311	4.22 ± 0.22	4.40 ± 0.18	0.065
Outer Perimeter/mm	5.15 ± 0.44	4.90 ± 0.31	0.137	5.20 ± 0.35	5.10 ± 0.34	0.468	5.46 ± 0.25	5.39 ± 0.17	0.505
Marrow Area/mm^2^	1.13 ±0.12	1.18 ± 0.18	0.501	1.02 ± 0.13	1.17 ± 0.17	**0.021**	1.18 ± 0.12	1.37 ± 0.12	**0.002**
Cortical Area/mm^2^	0.70 ± 0.10	0.60 ± 0.07	**0.009 6**	0.88 ± 0.06	0.74 ± 0.06	**6.72E-06**	0.91 ± 0.06	0.77 ± 0.03	**8.46E-06**
Total Area/mm^2^	1.84 ± 0.21	1.78 ±0.24	0.496	1.90 ± 0.18	1.91 ± 0.21	0.876	2.09 ± 0.16	2.14 ± 0.14	0.455
Trabecular									
BMC/mg	0.86 ± 0.25	0.64 ± 0.11	**0.017**	0.89 ± 0.11	0.74 ± 0.15	**0.012**	0.78 ± 0.12	0.63 ± 0.10	**0.008**
BMD/(mg/cc)	288.98 ± 58.05	247.43 ± 20.14	**0.044**	265.63 ± 24.75	218.67 ± 22.78	**5.64E-05**	216.09 ± 29.90	170.79 ± 24.00	**0.001**
TMC/mg	0.47 ± 0.21	0.30 ± 0.08	**0.026**	0.39 ± 0.10	0.25 ± 0.09	**6.56E-4**	0.25 ± 0.09	0.14 ± 0.06	**0.005**
TMD/(mg/cc)	553.41 ± 40.12	549.41 ± 14.69	0.768	545.17 ± 25.00	547.12 ± 24.63	0.846	569.09 ± 16.38	564.38 ± 20.40	0.564
BV/TV	0.28 ±0.09	0.21 ± 0.04	**0.040**	0.22 ± 0.05	0.13 ± 0.04	**1.51E-4**	0.12 ± 0.04	0.07 ± 0.02	**0.002**
BS/BV	45.46 ± 8.64	50.35 ± 2.80	0.103	45.07 ± 3.97	49.14 ± 3.96	**0.017**	46.37 ± 4.93	50.07 ± 5.78	0.130
Tb.Th/mm	0.05 ±0.01	0.04 ±0.002	0.073	0.04 ± 0.00	0.04 ± 0.00	**0.014**	0.04 ± 0.00	0.04 ± 0.00	0.132
Tb.N	6.00 ± 0.69	5.28 ± 0.75	**0.031**	4.83 ± 0.98	3.25 ± 0.90	**3.52E-4**	2.74 ± 0.65	1.61 ± 0.44	**2.58E-4**
Tb.Sp/mm	0.12 ± 0.03	0.15 ±0.03	**0.022**	0.17 ± 0.04	0.29 ± 0.12	**0.001**	0.34 ± 0.09	0.64 ± 0.27	**0.001**

Values in bold represent significant *P*-values for each comparison between WT and HBM OI mice

By four-point bending, femoral stiffness, yield load, and ultimate load were significantly decreased in HBM OI at all ages (Table 2). The dramatic 80%–90% decrease in post-yield displacement revealed that HBM OI bones are very brittle, snapping easily with little plastic (post-yield) deformation (*P* < 0.000 2).

**Table 2:** Biomechanics of HBM OI bone

	2 months WT	2 months HBM OI	*P*-value	6 months WT	6 months HBM OI	*P*-value	12 months WT	12 months HBM OI	*P*-value
Yield load/N	13.70 ± 4.89	11.05 ± 2.15	0.128	19.11 ± 3.59	11.94 ± 5.06	**5.23E-04**	20.80 ± 6.13	9.93 ± 2.65	**8.68E-05**
Ultimate load/N	21.02 ± 4.78	12.18 ± 2.05	**2.53E-05**	28.62 ± 3.28	13.38 ± 3.92	**3.47E-10**	30.22 ± 4.16	12.25 ± 2.83	**9.07E-10**
Stiffness/(N/mm)	131.12 ± 35.3	97.48 ± 24.07	**0.019**	201.73 ± 15.52	155.16 ± 31.40	**1.17E-04**	206.01 ± 27.78	148.77 ± 37.69	**7.38E-04**
Energy/N·mm	5.43 ± 3.25	1.04 ± 0.27	**1.77E-03**	5.85 ± 2.58	0.78 ± 0.29	**6.35E-06**	4.91 ± 1.80	0.65 ± 0.28	**1.11E-06**
Yield displacement/mm	0.12 ± 0.04	0.12 ± 0.02	0.986	0.11 ± 0.01	0.08 ± 0.03	**0.011**	0.11 ± 0.04	0.07 ± 0.01	**8.34E-03**
Ultimate displacement/mm	0.32 ± 0.09	0.15 ± 0.02	**8.14E-06**	0.27 ± 0.06	0.10 ± 0.02	**7.82E-09**	0.23 ± 0.04	0.09 ± 0.02	**9.62E-09**
Displacement ratio	2.84 ± 0.95	1.33 ± 0.32	**6.36E-04**	2.50 ± 0.80	1.16 ± 0.30	**8.29E-05**	2.32 ± 0.93	1.40 ± 0.41	**1.30E-02**
Failure load/N	20.52 ± 4.71	11.51 ± 2.10	**1.84E-05**	28.20 ± 3.08	13.11 ± 4.10	**4.9E-10**	29.57 ± 3.84	12.11 ± 2.82	**5.35E-10**
Failure displacement/mm	0.67 ± 1.14	0.15 ± 0.0	0.168	0.28 ± 0.08	0.10 ± 0.02	**5.35E-08**	0.25 ± 0.06	0.10 ± 0.03	**4.50E-07**
Elastic energy/N·mm	0.92 ± 0.39	0.65 ± 0.17	0.096	1.13 ± 0.26	0.64 ± 0.31	**6.98E-04**	1.31 ± 0.97	0.35 ± 0.14	**9.07E-03**
Plastic energy/N·mm	4.51 ± 3.10	0.39 ± 0.35	**2.04E-03**	4.72 ± 2.67	0.14 ± 0.19	**3.31E-05**	3.60 ± 1.93	0.29 ± 0.29	**6.61E-05**
Post-yield displacement/mm	0.23 ± 0.09	0.03 ± 0.03	**2.33E-06**	0.18 ± 0.08	0.02 ± 0.02	**9.52E-07**	0.14 ± 0.07	0.03 ± 0.03	**1.85E-04**
Bending moment/mm^4^	0.13 ± 0.03	0.11 ± 0.04	0.300	19.11 ± 3.59	11.94 ± 5.06	**5.23E-04**	20.80 ± 6.13	9.93 ± 2.65	**8.68E-05**
Predicted elastic modulus/MPa	591.71 ± 53.72	520.75 ± 129.27	0.178	28.62 ± 3.28	13.38 ± 3.92	**3.47E-10**	30.22 ± 4.16	12.25 ± 2.83	**9.07E-10**

Values in bold represent significant *P*-values for each comparison between WT and HBM OI mice

Patients with high bone mass OI have normal or increased bone mineral density on DXA.^19^^,^^22^^,^^25^^,27^ Femoral areal (2-dimensional) bone density of HBM OI mice was decreased at 2 months of age but recovered toward WT levels as mice aged (Fig. 3h) and was normal at age 2 years (Fig. S3C). Interestingly, vertebral areal bone density was normal at all ages examined (Fig. 3i, Fig. S3D). In comparison, tissue mineral density (TMD, 3-dimensional) was normal in both cortical and trabecular bone despite the bones’ extreme brittleness (Fig. 3j, k).

### HBM OI bone has increased calcium content with thinner and more disorganized mineral particles

**Fig. 4:**
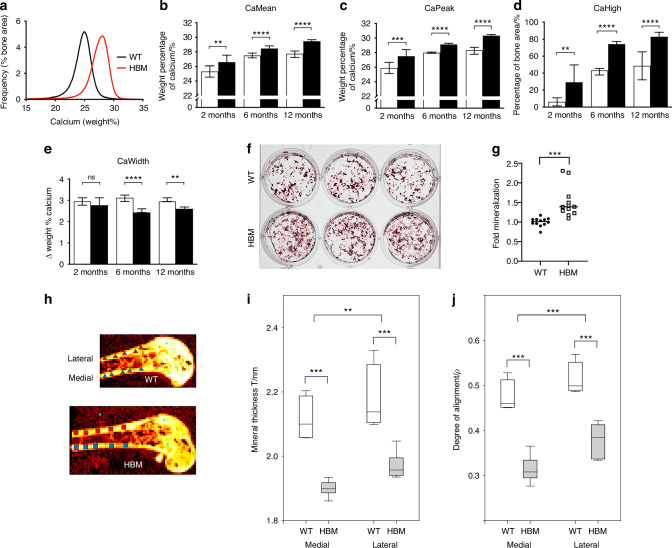
HBM OI femora are hypermineralized, with thinner, disorganized mineral particles. **a** Representative BMDD curve of 2-month-old WT and HBM OI long bone shows a distinct right-shift toward higher mineralization. **b**–**e** qBEI measurements of calcium content of WT and HBM OI femurs (*n* = 9–14 per age, per genotype). **b** CaMean, representing the mean calcium value, is increased in HBM OI at all ages. **c** CaPeak_,_ the most frequently measured calcium content, is increased in HBM OI bone at all ages. **d** CaHigh, representative of greater than the 95th percentile of the WT range, is significantly increased in HBM OI bone at all ages. **e** Bone matrix heterogeneity, measured by CaWidth, decreases in HBM OI as the mice aged, suggesting the presence of more uniform, mature bone than WT. **f** Representative Alizarin red staining of newborn calvarial osteoblasts differentiated in vitro for 28 days in culture displays increased mineral nodule formation (*n* = 5, each in triplicate). **g** Relative quantitation of alizarin red staining of calvarial osteoblasts (*n* = 12 wells per genotype). **h**–**j** Mineral particle thickness (T) and degree of alignment (ρ), in the medial (blue) and lateral (red) femoral cortex of HBM OI mice versus WT (*n* = 5–6 mice per genotype). Radiography of two typical bone sections is shown in (**h**) with the SAXS measurement positions indicated by colored symbols. Five measurements within the lateral and the medial cortex, respectively, were averaged for each animal. Statistical data based on an ANOVA test with repeated measures and Bonferroni pairwise comparisons are shown in (**i**) and (**j**). Both *T* and *ρ* are larger in the lateral cortex for all genotypes. These parameters are decreased in the HBM OI mice in both the medial and the lateral cortex. (WT = white, HBM OI = black/gray). (**P < 0.01, ***P < 0.005, ****P < 0.001)

HBM OI patients have increased bone tissue mineralization, higher than that present in classical OI bone.^19^ The distribution of calcium content in HBM OI murine bone was dramatically shifted toward higher values compared to WT at 2 months of age (Fig. 4a). CaMean and CaPeak were significantly higher in HBM OI compared to WT (Fig. 4b, c), demonstrating increased matrix mineralization at all ages. Moreover, while peak calcium levels increased in WT as well as in HBM OI bone from 2- to 6-months (both: *P* < 0.001), in WT peak calcium plateaued at 6 months (*P*-value versus 12 months: 0.889), whereas HBM OI increased from 6- to 12-months (*P* = 0.012; based on ANOVA test with Bonferroni pairwise comparisons). Furthermore, CaHigh was markedly increased (4.7-fold) in 2-month-old HBM OI mice, remaining significantly higher than WT as mice aged (Fig. 4d). The decrease in CaWidth in HBM OI is indicative of a more homogeneous, highly mineralized bone (Fig. 4e).

Corroborating the increased overall mineralization in the bone tissue, calvarial HBM OI osteoblasts differentiated for 28 days in culture deposited significantly more mineral (Fig. 4f, g). In contrast, homozygous HBM OI/HBM OI calvarial osteoblasts deposited significantly less mineral than cells from HBM OI or WT littermates (Fig. S2G, H), although it has a more severe phenotype. The decrease in mineral may be due to a more homogeneous matrix; nevertheless, the increased severity of these mice shows that mineral composition is not the only contributing factor.

Organization of the increased bone mineral content was analyzed by small-angle X-ray scattering (SAXS, Fig. 4h–j). Thickness (*T*) and degree of alignment (ρ) of mineral particles of the femoral cortex were reduced in HBM OI bone, which had thinner and less well-aligned mineral particles than WT, with smaller *T* and ρ in the medial vs the lateral cortex (all comparisons, *P* < 0.001).

### HBM OI has decreased cortical and trabecular bone with increased osteoid

Trabecular bone volume (BV/TV) of HBM OI femora was significantly decreased (33%, *P* = 0.005) in static histomorphometry, as was cortical width (Ct.W, 20%), in agreement with μCT (Table 3). The decreased volume reflects decreased trabecular number (Tb.N., 31%), rather than altered trabecular thickness. Osteoblast number (Ob.N) and number of osteoblasts on the bone perimeter (Ob/B.Pm) were normal, as was bone formation rate (BFR/BS). Unmineralized osteoid was significantly increased on HBM OI bone surface (OS/BS, 6.1-fold) (Fig. S3E), in both volume (OV/BV, 6.25-fold) and thickness (O.Th, 1.3-fold), with a concomitant increase in mineralization lag time (Mlt, 11.3-fold). Although total osteoclast number (N.Oc) and eroded surface/bone surface (ES/BS) were normal, the number of osteoclasts on the bone perimeter (N.Oc/B.Pm, 1.8-fold) was increased. Furthermore, both genotypes showed similar rates of new bone formation, or mineral apposition rate (MAR). However, the adjusted apposition rate (Aj.AR), which reflects the MAR in relationship to the mineralizing surface per osteoid surface, was significantly reduced (35%), due to the large amount of osteoid on the bone surfaces.

**Table 3:** HBM OI Histomorphometry

	Parameter	WT	HBM OI (*n*=10)	*P*-value
Static	(BV/TV)/%	6.15 ± 1.71 (*n* = 9)	4.14 ± 0.93	**0.005**
	(OV/TV)/%	0.04 ± 0.02 (*n* = 9)	0.25 ± 0.14	**0.000 4**
	(BS/TV)/(mm^2^/mm^3^)	3.96 ± 0.69 (*n* = 9)	2.72 ± 0.45	**0.000 2**
	(OS/BS)/%	3.13 ± 1.16 (*n* = 9)	19.05 ± 7.84	**0.000 01**
	(Ob.S/BS)/%	1.57 ± 0.71 (*n* = 9)	4.46 ± 3.09	**0.01**
	Tb.Th/µm	30.73 ± 3.69 (*n* = 9)	31.39 ± 3.92	0.85
	O.Th/µm	3.65 ± 0.84 (*n* = 9)	4.74 ± 0.80	**0.01**
	Tb.Sp/µm	480.60 ± 79.77 (*n* = 9)	724.78 ± 129.53	**0.000 2**
	Tb.N/mm^−1^	1.98 ± 0.35 (*n* = 9)	1.36 ± 0.23	**0.000 2**
	(N.Oc/B.Pm)/mm	0.80 ± 0.56 (*n* = 9)	1.42 ± 0.62	**0.000 2**
	N.Oc	6.89 ± 4.99 (*n* = 9)	7.60 ± 3.27	0.71
	(N.Ob/B.Pm)/mm	75.25 ± 17.84 (*n* = 9)	85.05 ± 18.90	0.28
	N.Ob	12.78 ± 4.12 (*n* = 9)	27.67 ± 11.24	**0.05**
	Ct.W/µm	241.85 ± 36.46 (*n* = 9)	192.66 ± 36.63	**0.009**
	(ES/BS)/%	0.34 ± 0.26 (*n* = 9)	0.42 ± 0.37	0.61
Dynamic	BFR/BS	0.59 ± 0.23 (*n* = 10)	0.42 ± 0.26	0.15
	Mlt	0.27 ± 0.19 (*n* = 10)	3.05 ± 2.68	**0.004**
	MAR	1.47 ± 0.38 (*n* = 10)	1.56 ± 0.57	0.62
	Aj.AR	39.68 ± 8.25 (*n* = 10)	25.80 ± 7.82	**0.000 4**

Values in bold represent significant *P*-values for each comparison between WT and HBM OI mice

Levels of serum bone turnover markers corroborated the histomorphometry results. TRAcP levels, a marker of bone resorption, were significantly increased in HBM OI mice (Fig. S3F), in agreement with an elevation of bone turnover. Additionally, PINP levels were normal in HBM OI, corresponding with a normal BFR/BS (Fig. S3G).

### HBM OI bone has decreased extracellular matrix with altered collagen crosslinks, and affects the osteoblast cytoskeleton

**Fig. 5:**
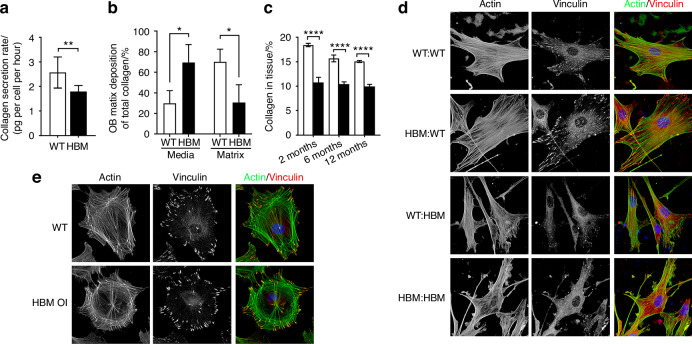
HBM OI mice have diminished collagen matrix with altered actin filaments and focal adhesions. **a** The rate of collagen secretion per cell into conditioned medium by undifferentiated osteoblasts is decreased in HBM OI, as measured by picrosirius red and normalized to cell count (*n* = 3, run in triplicate). **b** In 2-week post-confluent osteoblast cultures, less collagen is incorporated into the extracellular matrix in HBM OI cultures, as compared to WT (*n* = 3). **c** Femora at 2-, 6-, and 12-months of age consistently have a decreased amount of collagen in tissue by mass spectrometry (*n* = 3–4 per genotype at each age). **d** Immunofluorescence microscopy of WT and HBM OI osteoblasts plated on WT or HBM OI decellularized matrix, written as osteoblast genotype:matrix genotype. Cells grown on WT matrix show central actin stress fibers and focal adhesion puncta, while cells grown on HBM OI matrix have fewer central stress fibers and fewer central focal adhesions, and the presence of lamellipodia (*n* = 3). **e** Immunofluorescence microscopy of WT and HBM OI osteoblasts plated on glass slides. Both genotypes have well-spread cells with similar actin and vinculin staining (*n* = 2). (green = actin/phalloidin, red = vinculin, blue = DAPI, yellow = overlay of actin/vinculin) (*P < 0.05, **P < 0.01, ***P < 0.005, ****P < 0.001)

The disruption of the C-propeptide cleavage site may alter helical folding. HBM OI osteoblast procollagen chains, however, were found to form triple helices at a normal rate (Fig. S4A). Additionally, pepsin-cleaved type I collagen chains from either fibroblasts or osteoblasts show no difference in migration on SDS-urea-PAGE (Fig. S4B), suggesting that post-translational modification and folding rate are unaltered. Nevertheless, HBM OI osteoblasts show a 30% decrease of secreted procollagen in vitro (Fig. 5a). In addition, the percentage of secreted collagen deposited into the extracellular matrix in vitro was lower from HBM OI than WT osteoblasts (40% vs 70% incorporated, respectively) (Fig. 5b). Decreased collagen secretion and mutant collagen incorporation into bone matrix correlate with decreased femoral collagen content (59%–66% of WT, Fig. 5c). Collagen crosslinks are altered in HBM OI bone, with increased hydroxylpyridinoline (HP) trivalent crosslinks at 2- and 12-months and increased lysylpyridinoline (LP) crosslinks at 2 months, but without a significant change in the HP/LP ratio (Fig. S4C–E).

As an altered extracellular matrix can affect cellular function, we plated osteoblasts on WT or HBM OI decellularized, mineralized matrix in vitro. WT osteoblasts plated on WT matrix showed long actin fibers and multiple vinculin contact points anchoring the cells to the matrix (Fig. 5d). The cytoskeleton of HBM OI osteoblasts plated on WT matrix appeared similar to that of WT. However, when either WT or HBM OI osteoblasts were plated on HBM OI matrix, vinculin staining appeared more diffuse in the center of the cell, remaining punctate along the outer edges. Actin fibers are stained most intensely around the cell periphery instead of across the cell center and appear to have active lamellipodia. To confirm this was a matrix effect, we plated osteoblasts directly on glass slides. Without an underlying matrix, organization of actin and vinculin was similar in WT and HBM OI osteoblasts, with actin fibers and vinculin contacts across the cell center and along the periphery (Fig. 5e).

### Upregulation of osteoblast differentiation markers in HBM OI osteoblasts

**Fig. 6:**
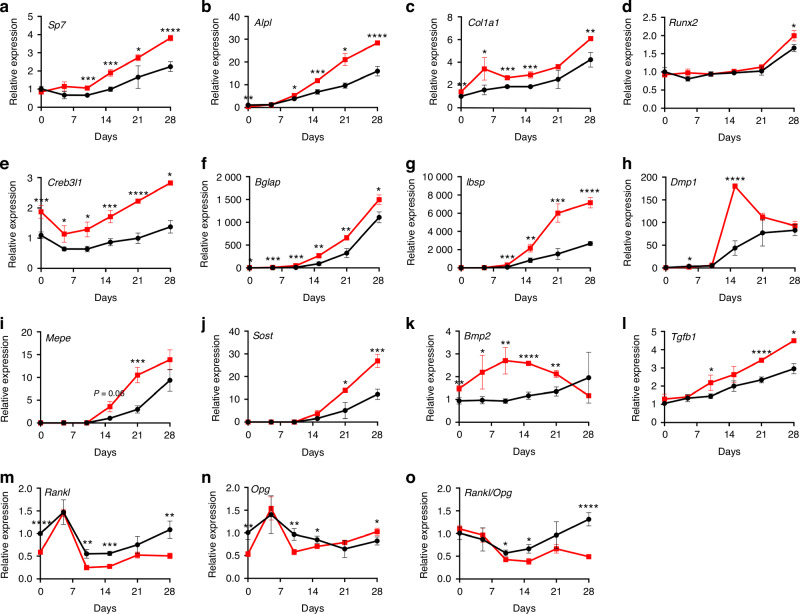
Osteoblast differentiation and ossification gene expression are increased in HBM OI osteoblasts. Representative qPCR expression levels from pooled calvarial osteoblast preps (*n* = 3–4) showing changes in early, mature, and late osteoblasts. Transcripts of early osteoblast markers **a** osterix (*Sp7)*, **b** alkaline phosphatase (*Alpl)*, and **c** type I collagen (*Col1a1)* are increased in HBM OI osteoblasts. **d** Transcripts of early osteoblast marker *Runx2* are slightly increased only at day 28 of differentiation. Transcripts of mature osteoblast markers **e** OASIS (*Creb3l1*), **f** osteocalcin (*Bglap*), and **g** bone sialoprotein (*Ibsp*) are increased in HBM OI osteoblasts. Transcripts of late osteoblast/osteocytes markers **h** dentin matrix protein (*Dmp1*), **i** matrix extracellular phosphoglycoprotein (*Mepe*), and **j** sclerostin (*Sost*) are increased in HBM OI osteoblasts compared to WT. **k** Upregulation of bone morphogenetic protein 2 (*Bmp2*) and **l** transforming growth factor beta (*Tgfb1*) expression may increase osteoblast differentiation. Transcripts of **m** RANK ligand (*Rankl/Tnfsf11*) and **n** osteoprotegerin (*Opg/Tnfrsf11b*) lead to a decreased **o** Rankl/Opg ratio in HBM OI osteoblasts. (WT = black, HBM OI = red) (*P < 0.05, **P < 0.01, ***P < 0.005, ****P < 0.001)

Cytoskeletal changes can affect osteoblast differentiation.^28^ In HBM OI differentiated osteoblasts, immature osteoblast markers were increased (*Sp7, Alpl, Col1a1)* (Fig. 6a–c) except for *Runx2*, which was increased only at day 28 (Fig. 6d). The increased *Col1a1* expression may be driven by an upregulation of *Creb3l1* transcripts, encoding the collagen transcription factor OASIS (Fig. 6e). Mature osteoblast (*Bglap, Ibsp*; Fig. 6f, g) and osteocyte *(Dmp1, Mepe, Sost;* Fig. 6h–j) transcripts were also significantly increased. *Wnt* and *Tgfb1* transcripts, encoding promoters of osteoblast differentiation, were significantly increased, potentially contributing to the increased osteoblast differentiation (Fig. 6k, l). Furthermore, expression of osteoclastogenesis regulators RANK ligand (*Rankl*/*Tnfsf11)* and osteoprotegerin (*Opg/Tnfrsf11*) was lower in HBM OI than in WT, and the Rankl/Opg ratio was decreased in HBM OI osteoblasts during late differentiation (Fig. 6m–o).

### Upregulation of ossification genes and downregulation of cell adhesion genes are most pronounced at the start of mineralization

**Fig. 7:**
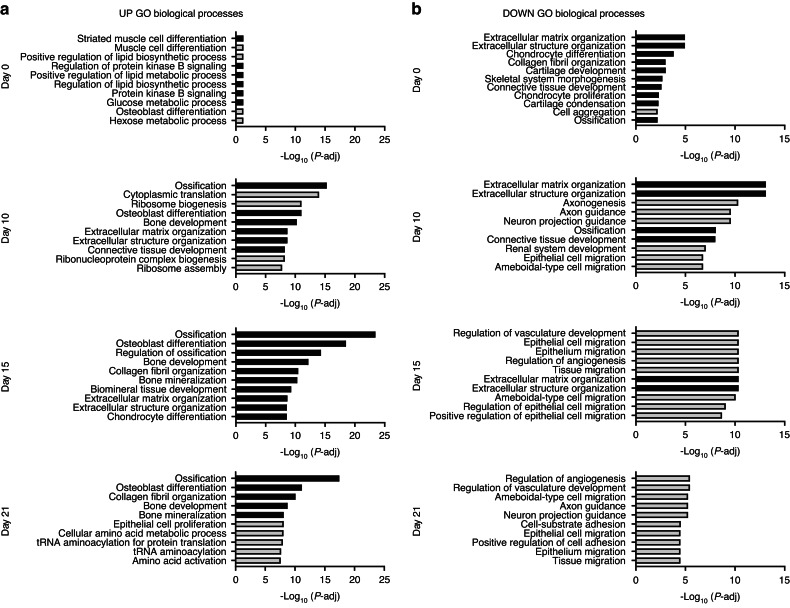
Upregulation of ossification and downregulation of cellular adhesion pathways in HBM OI mice. **a** Gene ontology (GO) analysis of the top ten biological processes upregulated in HBM OI osteoblasts during pre-differentiation (Day 0) and after 10, 15, and 21 days of differentiation. Ossification is the top upregulated pathway in HBM OI on days 10–21. Black-filled bars represent pathways related to bone and extracellular matrix. **b** GO analysis of the top ten downregulated biological processes in HBM OI osteoblasts pre-differentiation (Day 0) and after 10, 15, and 21 days of differentiation. Organization of the extracellular matrix is one of the top-downregulated pathways on days 0–15. Black-filled bars represent pathways relating to bone and extracellular matrix

To expand our understanding of how impaired C-propeptide cleavage affects osteoblast gene expression, RNA from pooled newborn calvarial osteoblast cultures was analyzed by RNA-Seq both at confluence (Day 0) and after 10, 15, or 21 days of differentiation. Gene ontology (GO) analysis revealed that pathways involved in ossification, bone development, and bone mineralization were highly enriched in HBM OI osteoblasts (Fig. 7a). Ossification was the top upregulated pathway at days 10, 15, and 21. Transcripts involved in Wnt signaling were also increased; these included osteoprogenitor markers (*Lgr6*, *Gli1*, *Tcf7*, *Wnt10b*) and Wnt/BMP inhibitors (*Sost*, *Sostdc1*, *Ccdc88c*, *Bambi*, *Dkk1*). Interestingly, the top-downregulated GO pathways involved cell adhesion, cell migration, and angiogenesis (Fig. 7b), all of which are related to the extracellular matrix. Days 10 and 15 of differentiation had the highest number of differentially expressed genes (DEG), most of which were upregulated (51/82 and 76/99 genes, respectively) (Table S1).

**Fig. 8:**
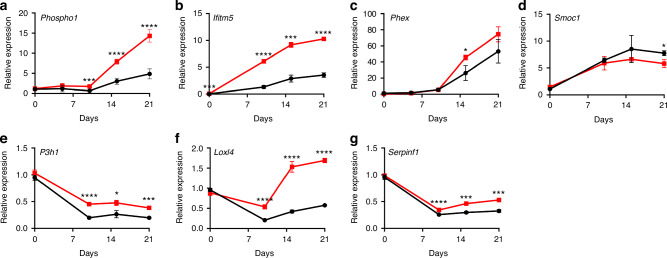
Validation of increased ossification gene expression and altered expression of extracellular matrix genes. qPCR confirmations were performed on calvarial osteoblasts to validate the RNA-Seq data. Expression of ossification transcripts **a** phosphoethanolamine/ phosphocholine phosphatase 1 (*Phospho1*), **b** interferon-induced transmembrane protein 5 (*Ifitm5*), and **c** phosphate-regulating endopeptidase X-linked (*Phex*), was confirmed to be increased starting at days 10–15 of differentiation. **d** Transcripts of extracellular matrix binding protein SPARC Related Modular Calcium Binding 1 (*Smoc1*) are decreased in HBM OI osteoblasts at Day 21. An increase in transcripts of collagen-interacting proteins **e** prolyl 3-hydroxylase 1 (*P3h1*), **f** lysyl oxidase-like 4 (*Loxl4*), and **g** serpinF1 (*Serpinf1*), corroborates the RNA-Seq data. (WT = black, HBM OI = red) (*P < 0.05, **P < 0.01, ***P < 0.005, ****P < 0.001)

Both upregulated and downregulated genes were enriched for many GO pathways, including mineralization, osteoblast differentiation, and extracellular matrix. Upregulated mineralization (*Smpd3*, *Phospho1* (Fig. 8a), *Sgms2*, *Bglap* (Fig. 6f), *Ibsp* (Fig. 6g), *Ifitm5* (Fig. 8b), and *Phex* (Fig. 8c)) and osteoblast differentiation genes *(Tnn, Bmp8a, Bmp3, Sp7* (Fig. 6a), and *Shox2*) encode proteins which promote mineral deposition while downregulated ossification transcripts involved endochondral bone formation and Wnt signaling (*Comp*, *Matn1*, *Fzd9*, *Nell1*, *Wnt11*, *Rspo2, Trp63, Igf2, Smoc1* (Fig. 8d)). Extracellular matrix GO terms were enriched in upregulated genes encoding collagen-interacting proteins, such as *P3h1* (Fig. 8e), *Adamts18*, *Loxl4* (Fig. 8f) and *Serpinf1* (Fig. 8g) while downregulated extracellular matrix pathways included cartilage-related (*Cnmd*, *Col2a1*, *Col9a1*, *Col9a2*, *Col9a3*, *Cytl1*) and adhesion genes (*Itga2*, *Meltf*, *Hmcn1*, *Tenm1*, *Fam107a*, *Thbs4*, *Col26a1*). The decrease in cell migration and angiogenesis gene pathways might both impact matrix organization and be secondarily and reciprocally influenced by the quantity and structure of collagen in the matrix (Fig. 7b).

### Alterations in HBM OI osteocytes may influence gene expression

Giemsa staining of 2-month-old femurs revealed that fewer osteocyte lacunae were present in HBM OI cortical bone (Fig. S5A). In addition, osteocytes were visually less aligned with lamellae than in WT. Quantitative backscattered electron imaging (qBEI) of osteocyte lacunar sections (OLS) confirmed that HBM OI bone had significantly fewer osteocyte lacunae than WT at 2-, 6-, and 12-months (decreased 19%–35%, Table S2), resulting in decreased bone micro-porosity at 2- and 6-months (14%–21%). Osteocyte lacunae are larger in area in HBM OI than in WT bone, with a slight increase in perimeter at 2- and 12-months, but their shape (OLS ratio) was unchanged.

Although primary osteoblast function can be investigated in culture, bone tissue primarily contains osteocytes and is more representative of osteocyte function. RNA from 2-month femoral HBM OI bone revealed decreased expression of *Col1a1*, *Bglap*, and *Sost*, opposite to cultured newborn calvarial osteoblasts (Fig. S5B). These decreases in expression may reflect what is happening in the bone tissue itself; *Sost* expression in the femur matches the reduced osteocyte number on histology, and *Col1a1* expression reflects the lowered collagen content in bone tissue (Fig. 5c). In contrast, the genes involved in osteoclast differentiation (*Acp5*, *Ctsk*, *Rankl/Opg*) were increased in the femur, aligning with increased TRAcP levels in HBM OI serum (Fig. S3F).

### Human HBM OI bone has increased osteoid, bone density, and mineralization in adulthood

**Fig. 9:**
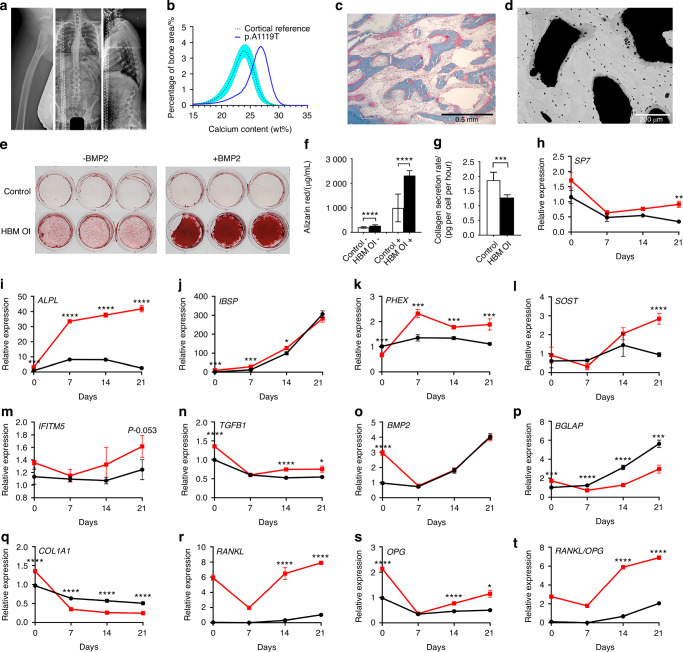
Human HBM OI adult phenotype. **a** Radiographs of the COL1A2 p.A1119T patient at 26 years old. Left panel: Right femur showing thick cortices, Middle panel: AP spine with minimal curvature; Right panel: Lateral spine with normally shaped vertebrae. **b** BMDD curve of cortical area from radial bone of the HBM OI patient compared to cortical transiliac bone reference controls. **c** Goldner’s trichrome staining of HBM OI cortical bone showing an increased amount of osteoid (red) along the bone surface (blue). **d** Representative backscattered electron image of the patient's cortical bone used for measuring osteocyte parameters. **e** Alizarin red staining of α2(I) p.A1119T primary osteoblasts differentiated in vitro for 6 weeks in culture displayed an increase in mineral nodule formation with or without BMP2 treatment. (*n* = 2) **f** Quantitation of alizarin red staining of α2(I)p.A1119T osteoblasts (*n* = 2, in triplicate). **g** Collagen secretion rate by patient osteoblasts was decreased by approximately 30% as compared to age-matched control osteoblasts (*n* = 2, in triplicate). Representative qPCR expression levels from differentiating osteoblast cultures (*n* = 2) showing changes in early, mature, and late osteoblasts. Transcripts of early osteoblast markers **h** osterix (*SP7)*, and **i** alkaline phosphatase (*ALPL)* are increased in human HBM OI osteoblasts. Transcripts of osteoblast markers **j** bone sialoprotein (*IBSP*), **k** phosphate-regulating endopeptidase X-linked (*PHEX*), **l** sclerostin (*SOST)* are increased in human HBM OI osteoblasts, comparable to expression in murine osteoblasts. **m** *IFITM5* transcripts were increased in human HBM OI, similar to mouse, but did not reach significance. **n** *TGFB1* expression is increased on days 0, 14, and 21 of differentiation. **o** *BMP2* expression is significantly increased in undifferentiated osteoblasts (day 0), then normalizes during differentiation. **p** Osteocalcin (*BGLAP*) and **q** type I collagen (*COL1A1*) transcripts are decreased starting at day 7 of differentiation. Transcripts of **r** RANK ligand (*RANKL/TNFSF11*) and **s** osteoprotegerin (*OPG/TNFRSF11B*) are prominently increased, leading to an increased RANKL/OPG ratio (**t**) in HBM OI osteoblasts, indicating intense bone turnover. (WT = black, HBM OI = red) (*P < 0.05, **P < 0.01, ***P < 0.005, ****P < 0.001)

We previously described the first HBM OI proband (P2),^19^ with a *COL1A2* p.A1119T substitution, when he was 12 years of age. He is currently 27 years old (updated clinical report in Supplementary Clinical Information). On radiographs, his lower extremities showed evidence of mild osteopenia, while femur cortices remained thickened, but not osteopetrotic (Fig. 9a, left panel). He has minimal thoracic scoliosis and mild to moderate facet arthropathy in the lower lumbar spine (Fig. 9a, middle, right panels).

Bone samples obtained after surgery of a radial fracture non-union were examined by qBEI and histomorphometry; primary osteoblasts were cultured from bone chips.^29^ As at age 12, bone mineralization density distribution (BMDD) of bone at age 27 years showed increased CaMean, CaPeak, and CaHigh as compared to transiliac cortical reference controls (Fig. 9b, Table S3). Bone tissue showed increased osteoid and increased osteocyte porosity, with a larger lacunar area and perimeter, but a normal OLS density (Fig. 9c, d, Table S3). In contrast to HBM mice, the shape of the lacunae was more round vs reference values (OLS-aspect ratio decreased).

### Human HBM OI osteoblasts have increased expression of mineralization and osteoclastogenesis genes

Primary HBM OI osteoblasts derived from bone chip outgrowth reflect more mature bone cell populations than osteoblasts from neonatal calvarial preps. These cells were differentiated for 21 days and compared to age- and gender-matched control osteoblasts. HBM OI osteoblasts displayed a significant increase in mineral deposition compared to control, both with and without BMP2 treatment (Fig. 9e, f). Like murine HBM OI, proband osteoblasts have ~30% decreased collagen secretion (Fig. 9g). As with HBM OI murine osteoblasts, genes involved in mineralization and osteoblast differentiation (*SP7, ALPL, IBSP, PHEX*, *SOST)* (Fig. 9h–l) were increased. Notably, *PHEX* (about 2-fold) and *ALPL* (approximately 40-fold) transcripts are increased in HBM OI, the products of which promote mineralization by breaking down mineralization inhibitors. *IFITM5* transcripts (Fig. 9m) trended higher in HBM OI but did not reach significance. *TGFB1* transcripts were elevated (Fig. 9n), while *BMP2* expression was increased 3.1-fold at day 0 but was normal during differentiation (Fig. 9o). While both osteocalcin (*BGLAP*) and type I collagen (*COL1A1*) expression levels were increased in undifferentiated osteoblasts, transcripts were reduced starting at differentiation day 7 (Fig. 9p, q). These results agree with the decrease of these genes in 2-month-old HBM OI femoral bone (Supplemental Fig. 5B). *RANKL/TNFSF11* was 6- to 8-fold higher in adult HBM OI *COL1A2* p.A1119T cells (Fig. 9r). *OPG/TNFRSF11* expression was also elevated; nonetheless, the *RANKL/OPG* ratio was 2.75- to 7-fold higher throughout osteoblast differentiation (Fig. 9s, t).

## Discussion

Mutations causing OI have provided unexpected and important insights into the role of type I procollagen C-propeptide in bone formation. First, it was unanticipated that missense mutations in the C-propeptide of either proα1(I)/proα2(I) would cause moderate to severe OI, because the C-propeptide is processed before the incorporation of type I collagen into the extracellular matrix.^16,30^ These substitutions can impact chain assembly, interchain disulfide bonds, and procollagen ER localization.^16,17,18^ Second, and more surprisingly, substitutions in any of the residues constituting the type I procollagen C-propeptide cleavage site cause HBM OI with elevated DXA bone mass and moderate fragility.^19^ Adults with HBM OI have dense bones with extreme mean calcium content and scalloped edges, raising issues of increasing mineralization over time and potential osteopetrosis.^27^ With few case reports,^22,25,27^ fundamental issues about the function of type I pC-collagen in bone, and the mechanism of this dysplasia are poorly understood.

To investigate the differences between HBM OI and classical OI, and especially the paradoxical high bone mass aspect of HBM OI, we generated an HBM OI murine model, in which both the alanine and aspartic acid residues of the α1(I) C-propeptide cleavage site have been substituted. While the substitution of both C-propeptide cleavage site residues in one allele could lead to a more severe phenotype than in HBM OI patients (who only have a single residue of the cleavage site affected), nevertheless the HBM OI mouse phenotype has the same overall characteristics to that of HBM OI individuals, with fragile bones, a normal DXA bone density (by 12 months of age), and excess bone hypermineralization. The HBM OI mouse data establishes that the proper timing, site, and localization of C-propeptide cleavage are critical for skeletal tissue and cells and strongly impact bone mineralization.

pC-collagen, in which the C-propeptide is attached to the collagen helix, can form fibrils with C-propeptides located at gap/overlap junctions.^31,32^ In murine HBM OI skin, incorporation of pC-collagen inhibited lateral aggregation of the fibril, resulting in smaller fibrils with more uniformly-sized diameters, similar to the *COL1A2* p.A1119T patient.^19^ In HBM OI bone fibrils, pC-collagen incorporation led to a “barbed-wire” appearance, as seen in mice and patients lacking BMP-1.^33^ The *Bmp1*^−^^*/*^^−^*/Tll1*^−^^*/*^^−^ mouse lacks C-propeptide cleavage in bone,^34,35^ suggesting there is no cleavage redundancy in bone from other enzymes, such as meprins,^36^ and amplifying the effect of impaired cleavage in bone. We found a substantial amount of C-propeptide monomer in HBM OI osteoblast matrix and bone lysates vs WT, supporting eventual propeptide cleavage in bone tissue. BMP-1 cleaves α1(III) procollagen at a Gly-Asp dipeptide,^37^ rather than the Ala-Asp of α1(I) collagen. There is presently no support for BMP-1 cleavage of type I procollagen at a Gly-Asp dipeptide 46 nucleotides upstream of the canonical site in the collagen helical region, since BMP-1 requires both a correct target sequence and a proper tertiary configuration. Alternatively, the excess C-propeptide could be released by cathepsin K, which can cleave type I collagen at multiple sites, including the C-telopeptide,^38^ during bone remodeling and remain in the matrix.

The hallmark features of HBM OI are a normal to high areal bone density in combination with tissue mineralization by qBEI that exceeds the standard hypermineralization of classical OI.^19^ HBM OI murine and human cultured osteoblasts deposit a striking 1.5–3-fold increase in mineral vs control. Two-month-old HBM OI mice have low femoral DXA bone density but attain normal bone density by 12 months. In contrast, BMDD of murine HBM OI bone showed substantial increases in CaMean, CaPeak, and especially CaHigh, confirming the hypermineralization of HBM OI bone (Fig. 4), in excess of BMDD results in classical OI murine models.^39^ HBM OI bone had some similarity to the atypical type VI OI mouse, which also has a mineralization defect, though HBM OI had a greater effect on CaHigh vs its WT control.^40^ Adult and pediatric HBM OI individuals or those with recessive *BMP1* mutations had the highest CaMean levels reported on BMDD.^27^ Patients with type VI OI, caused by recessive null *SERPINF1* mutations, also had higher CaMean and CaHigh than classical OI.^41^ While hypermineralization in classical OI results from higher density packing of normal-size mineral particles,^42–44^ the thinner mineral particles in HBM OI revealed by SAXS may allow more particles to be packed into the same area.

The extensive hypermineralization of HBM OI bone may also be related to increased mineralization homogeneity in fibrils. Mineral particles generally co-align with collagen fibrils,^45–47^ whose imperfect alignment in HBM OI likely contributes to the misalignment of mineral particles. As mineral particles are normally deposited heterogeneously in the gap/overlap region of fibrils,^31,48^ there may be physical obstruction from the uncleaved C-propeptide preventing the normal localization of minerals, and leading to mineralization elsewhere. We hypothesize that decreased mineral alignment reflects disorganization of the collagen matrix in HBM OI and that increased mineralization relates to denser mineral packing within and along the collagen fibrils.

Without proper alignment of mineral crystals, cracks propagate, rather than being deflected, which is why well-aligned lamellar bone is especially tough.^49^ The need for continual microcrack damage repair correlates with increased remodeling found in murine and human HBM OI bone. Also, elevated TRAcP levels and increased *Acp5* and *Ctsk* transcripts in murine HBM OI femur, despite normal osteoblast and osteoclast numbers, speak to increased osteoclast resorption. Osteoblasts from our adult *COL1A2* p.A1119T HBM OI patient show up to ~7-fold increase in *RANKL/TNFSF11* transcripts, indicating high osteoclast stimulation. Additionally, Shu and others showed that osteoblasts grown on microcracked hydroxyapatite had increased maturation, alkaline phosphatase activity, and mineral deposition, much like HBM OI.^50^

Murine HBM OI long bones are extremely brittle, with an approximately 90% decrease in post-yield displacement vs. WT, as well as decreased stiffness, yield, and ultimate load. The *Bmp1*^−^^*/*^^−^*/Tll1*^−^^*/*^^−^ postnatal KO mouse shows similar decreases in post-yield displacement and maximum load,^34^ supporting the lack of C-propeptide processing as causing decreased bone quality. However, slower type I collagen cleavage alone does not lead to bone brittleness. The *Pcolce*^−^^*/*^^−^ mouse, lacking the C-propeptidase enhancer protein PCPE1, does not have brittle bones, but rather an adaptive geometry.^51^ Instead, C-propeptide presence in matrix appears to be critical, since C-propeptide staining was present on immunofluorescence microscopy of collagen fibrils deposited by *Bmp1*^−^^*/*^^−^*/Tll1*^−^^*/*^^−^ MEFs but was absent in similar studies of *Pcolce*^−^^*/*^^−^ collagen fibrils from matrix deposited in vitro.^51^

Human HBM OI long bone does not appear to be as fragile as murine HBM bone. While HBM OI patients do have childhood fractures, they have fewer long bone fractures than most cases of classical OI, with a phenotype more similar to type I or mild type IV OI.^19,27^ The HBM OI collagen mutation would be expected to affect both long bone (with mechanics dominated by cortex) and the more trabecular vertebral bone. The lumbar spine DXA z-scores of HBM OI individuals range from −2.6 to +7.7, with vertebral fractures noted in some patients.^19,25^ Our patient has thumbprint vertebral compressions but not vertebral fractures. The organization of the increased mineral content in the bone may result in a greater (in HBM) or lower (in classical OI) density on 2-D measurements of bone density. pQCT volumetric measurement of the lumbar spine in our patient shows decreased z-scores (−1.8 to −2.0) and would likely better reflect the true density of the bone in these patients. Vertebral DXA z-scores of HBM OI mice were normal, but neither volumetric bone density nor crush experiments were performed to measure their brittleness.

Murine and human HBM OI bone,^19^ and bone from patients with BMP1 mutations,^52,53^ have thick osteoid seams, indicating defective mineralization timing. Human type VI and atypical type VI OI, caused by recessive null *SERPINF1/*PEDF mutations and a dominant *IFITM5* p.S40L mutation, respectively, also display an increased osteoid in bone tissue.^54–56^ Nevertheless, HBM OI and type VI OI have strikingly different phenotypes. In type VI OI, individuals are normal at birth but progress to a severe clinical phenotype with age, with a high fracture rate, bowed long bones, vertebral compressions, severe scoliosis, barrel chest, and short stature.^55^ HBM OI has a generally mild to moderate phenotype with few fractures, without bowing and late development of moderate scoliosis, suggesting that the histological mineralization delay in these OI types either has different mechanisms, or is not crucial to the phenotype. PEDF impacts mineralization by binding to the PEDF receptor and regulating osteocyte gene expression through ERK signaling,^57^ while HBM OI bone has excess C-propeptide in the matrix. Currently, no evidence suggests that the presence of the C-propeptide or pC-collagen can alter ERK activation.

Excess C-propeptide in tissue may also affect signaling pathways. Trimeric C-propeptide is involved in pathways such as osteoblast differentiation, cell attachment, and downregulation of collagen synthesis.^9,11,58,59^ Incomplete processing of α1(I) procollagen would decrease the C-propeptide available for signaling, despite upregulation of *Col1a1* expression in newborn murine HBM OI bone. Furthermore, altered vinculin staining of WT osteoblasts plated on HBM OI matrix implies cell attachment is affected. Cleaved C-propeptide has been shown to enhance TGFβ signaling in MC3T3 osteoblastic cells,^58^ amplifying the effect of increased latent TGFβ released from HBM bone due to high bone remodeling. TGFβ can promote increased osteoblast gene expression, alkaline phosphatase activity, and mineralization in MSCs co-stimulated with BMPs or when grown in proliferation media,^60^ much like the osteoblastic phenotype in HBM OI.

Functionality of propeptides from type I collagen and other extracellular matrix proteins has been demonstrated. Both type I and type II C-propeptides possess internalization sequences for entering cells.^61,62^ Exogenous ^125^I-labeled type I collagen C-propeptide was able to enter lung fibroblast nuclei and inhibit *COL1A1* transcription.^11^ Chondrocalcin, the cleaved C-propeptide of type II collagen, enters chondrocytes and binds the *Col2a1* promoter.^61,62^ Lysyl oxidase propeptide (LOX-PP) can bind proteins both inside the cell and in the matrix.^63^ LOX-PP functions as a tumor suppressor intracellularly and an inhibitor of FGF2 signaling extracellularly.^64^ We speculate that increased pC-collagen in bone tissue could lead to decreased C-propeptide internalization, resulting in less downregulation of *Col1a1* transcription compared to WT. It is unknown whether the C-propeptide released after pC-collagen deposition can enter cells.

The extracellular matrix produced by osteoblasts is the scaffolding on which bone mineral is deposited and also impacts cellular differentiation. Mineralization and differentiation of osteoblasts are consistently interdependent. Osteoblasts grown in vitro on type I collagen films vs plastic had enhanced differentiation and increased mineralization uniformity.^65^ Likewise, osteoblasts grown on roughened vs smooth titanium surface had increased maturation and BMP2 and BMP4 expression, with increased mineral deposition.^66^ Treatment of osteoblasts with Rho-associated coiled-coil containing kinase (ROCK) inhibitor showed increased osteoblastic differentiation and mineralization, enhanced osteoclastogenesis, and decreased cellular adhesion,^67,68^ changes similar to osteoblasts plated on HBM OI extracellular matrix. The loss of cellular adhesion due to altered extracellular matrix again correlates with increasing mineralization and may play a role in HBM OI bone. A decrease in the cellular adhesion to HBM OI matrix may be caused by the increase of collagen mineralization, as bone mineral would conceal integrin binding motifs on the collagen matrix surface, limiting adhesion sites.^69^

BMP-2 is a potent inducer of osteoblast differentiation and ectopic bone formation. The BMP-2-generated increase in osteoblast and osteoclast differentiation can be potentiated by TGF-β1.^70^ Thus, the increased *TGFB1* and *BMP2* expression in HBM OI may underlie the enhanced ectopic bone formation seen in the patellar and Achilles tendons of the HBM OI mice. Calcification of the Achilles tendon was also reported in some adult human HBM OI patients.^27^

In summary, murine HBM OI models many features of HBM OI patients (Table 4): it shares critical features, such as very high bone matrix mineralization, increased osteoid, a higher-than-expected areal bone density, and strikingly brittle bones. We can observe changes in the HBM OI mice throughout different life stages, and they can provide a platform for future drug studies. Importantly, the HBM OI mouse has led to new mechanistic insights, such as a few spontaneous fractures despite the severe brittleness of its bones, as well as the decrease in mineral crystal size and organization. Moreover, the presence of pC-collagen in bone fibrils and deposition into the matrix may lead to a more homogenous mineralization of fibrils, which can affect cell attachment, alter expression of mineralization genes, and enhance bone remodeling. Taken together, we have shown that the type I collagen C-propeptide and the proper timing of its processing impact bone development on a cellular and extracellular level and are critical for bone mineralization. Further research into the phenotypic role of extracellular matrix vs. signaling by cleaved C-propeptide was beyond the scope of this paper, but should be an important focus in future studies, which may lead to the identification of novel treatment strategies for this unique type of OI.

**Table 4:** Comparison between murine and human HBM OI

Parameters	HBM OI murine	Human HBM OI
Body Size	Smaller	Normal
Severity	Moderate	Mild
Osteoid	Increased osteoid volume and thickness	Increased osteoid in bone tissue
BMDD	Extremely high mineral content	Extremely high mineral content
Collagen processing	Decreased C-propeptide processing in fibroblasts and osteoblasts, pC-collagen, and C-propeptide found in bone tissue	Decreased C-propeptide processing by fibroblasts^19^
Fibrils	Smaller, more uniform skin fibrils, barbed-wire fibrils in bone	Smaller, more uniform skin fibrils^19^
Biomechanics	Very brittle	ND
µ-CT/pQCT	Decreased cortical and trabecular parameters	L1-L2 z = −1.8 (age 13)^19^ L1-L2 z = −2.0 (age 24)
DXA	Femur DXA decreased at 2 mo, increases to normal as mice age; normal vertebral DXA z-score	Whole bone *z* = +1.7 (age 27) L1-L4 *z* = +1.9 (age 27)
Extracellular matrix	Decreased amount, altered cell-matrix interactions	ND
Gene Expression	Increased mineralization and OB differentiation transcripts, decreased matrix and adhesion transcripts	Increased mineralization and OB differentiation transcripts
Osteocytes	Decreased osteocyte numbers, larger in area	Normal osteocyte numbers, larger in area

## Materials and methods

### Generation of HBM OI Mice

The two amino acid residues constituting the *Col1a1* C-propeptide cleavage site were substituted in BAC RP24-338I21 (Children’s Hospital Oakland Research Institute, Oakland, CA) containing the entire mouse *Col1a1* genomic sequence. For detailed information on clone generation, please see Supplementary Methods.

Chimeras generated from embryonic stem cells were backcrossed twice with EIIA-Cre in C57Bl/6 (The Jackson Laboratory, Bar Harbor, ME), then further backcrossed with WT C57Bl/6 for two additional generations to produce F4 mice. Genotyping of mice was performed on tail snip DNA extracted using the Extract-N-Amp PCR kit (Sigma-Aldrich, St. Louis, MO) and using the following primers: Geno-F: 5′-CGGTTATGACTTCAGCTTCCTGCC-3′ and Geno-R: 5′-AGGTCTGACCTGTCTCCA-TGTTGC-3′. Experiments were performed in accordance with an NICHD Animal Care and Use Committee-approved protocol.

For the retrieval of homozygous pups, heterozygous females underwent a C-section to remove pups at embryonic day 18.5, as all females who went to term developed dystocia. Pups were fostered by a dam in the colony with a recent litter.

### Skeletal staining

Skeletal staining was performed on newborn postnatal day 3 (P3) pups. Skin and organs were removed, and the skeletons were fixed in 95% ethanol for 7 days, followed by acetone for 7 days. Skeletons were stained for 3 days in 0.3% alcian blue plus 0.1% alizarin red at 37 °C, then destained with 1% KOH and increasing amounts of glycerol.

### Radiographs and bone density scans

Radiographs of mouse skeletons were captured on a Faxitron Ultrafocus^DXA^ imaging system (Hologic, Santa Clara, CA). Measurements of percent body fat and areal bone density of the L3–L4 vertebrae and the left femur were performed on a Piximus2 bone density scanner (*n* = 9–11, GE Lunar Corporation) for the adult mice or the Faxitron Ultrafocus^DXA^ imaging system for homozygous pups and their littermates.

### Growth Curves and measurements

Male mice of both genotypes were weighed once a week from 4–16 weeks of age, at 6- and 12-months (*n* = 9–16 mice per timepoint). Body and femur lengths were measured using digital calipers (Mitutoyo Corporation, Aurora, IL).

### Histology

Femurs from 2-month-old male mice and hind limbs from 6-month-old male mice were fixed in 70% ethanol. Femurs and limbs were embedded in paraffin and stained with Giemsa or Safranin O/Light Green (HistoServ, Germantown, MD). Slides were imaged on a Keyence BZ-X710 microscope (Keyence, Itasca, IL).

### Human subjects

A bone sample obtained as surgical discard from our HBM OI patient was received at NIH under an IRB-approved protocol (18-CH-0120) with informed consent.

### Cell culture

Primary murine fibroblasts were grown from abdominal skin explants from neonatal pups and cultured in Dulbecco’s Modified Eagle Medium (DMEM, Gibco, Gaithersburg, MD) supplemented with 10% fetal bovine serum (FBS, Benchmark, Gemini Bio-Products, West Sacramento, CA) and 1% Penicillin/Streptomycin (P/S, Gibco). Primary osteoblasts (OB) were isolated from newborn mouse calvaria between 2 days and 5 days after birth, except for OB from homozygous litters, which were isolated on the day of birth (E18.5). After washing calvaria with phosphate-buffed saline (PBS), the calvaria were digested with collagenase type II (200 U/mL, Worthington Biochemical, Lakewood, NJ) 5 times. Cells from digests 3–5 were pooled and plated. Osteoblasts were cultured in Minimum Essential Medium, Alpha (α-MEM, Gibco) supplemented with 10% FBS (GemCell, Gemini Bio-Products, West Sacramento, CA) and 1% P/S. Upon confluence (day 0), culture media were replaced with OB differentiation media, consisting of OB media supplemented with 100 μmol/L L-ascorbic acid (A7631, Sigma-Aldrich, St. Louis, MO) and 2.5 mmol/L β-glycerophosphate (G9891, Sigma). Differentiation media was changed three times per week for up to 28 days.

Human primary osteoblasts were prepared as previously described.^29^ In brief, bone chips were chopped into fine particles using a bone cutter and spring scissors, then digested with 0.3 U/mL Collagenase P (11213865001, Roche, Basel, Switzerland) for 2 h at 37 °C. Digested bone chips were plated in 10% α-MEM plus 50 µg/mL L-ascorbic acid and grown at 8% CO_2_, and cells were allowed to outgrow. Differentiation media for human osteoblasts was the same as for mouse, with supplementation of 10 nmol/L dexamethasone (D1756, Sigma-Aldrich) and with or without addition of 100 ng/mL BMP2 (355-BM, R&D Systems, Minneapolis, MN) for up to 6 weeks. The single bone sample available from an HBM OI patient yielded insufficient osteoblasts for robust comparison studies regarding the mechanism.

### Collagen assays

WT and HBM OI murine fibroblasts or osteoblasts were grown to confluence in 6-well culture dishes for all collagen assays. For the pericellular processing assay, confluent cells were serum-starved for 2 h in serum-free medium plus 50 µg/mL L-ascorbic acid, then labeled with 437.5 μCi/mL of 3.96 TBq/mmol L-[2,3,4,5-^3^H] proline (MT522E, Moravek, Brea, CA) in the same serum-free medium for 24 h, then chased with complete media containing 2 mmol/L cold proline. Media was collected with inhibitors (10 mmol/L Benzamidine (B6506, Sigma), 4 mmol/L EDTA (ED2SS, Sigma), 0.1 mmol/L N-ethylmaleimide (E3876, Sigma)) at the timepoints listed. Samples were precipitated with ammonium sulfate, and equal volumes were loaded on reduced 6% sodium dodecyl sulfate (SDS)-Urea-polyacrylamide gels (PAGE).

The chain incorporation assay was performed by serum-starving the cells for 2 h in serum-free medium plus 50 µg/mL L-ascorbic acid, labeling cells with 140 μCi/mL of 3.96 TBq/mmoL L-[2,3,4,5-^3^H] proline in serum-free media plus L-ascorbic acid for 80 min, then chasing with DMEM containing 10% FBS, 50 μg/mL L-ascorbic acid, and 10 mmol/L cold proline. Cell layer lysates were collected in PBS plus inhibitors (15 mmol/L iodoacetamide, 25.5 mmol/L EDTA, 6.7 μmol/L *N*-ethylmaleimide, 10 μmol/L phenylmethylsulfonyl fluoride (P7626, Sigma), and 2.2 nmol/L pepstatin (P4265, Sigma)) and ethanol precipitated. Procollagens were separated under non-reducing conditions by 5.5% SDS-Urea-PAGE.

Steady-state collagen analysis was conducted as previously described.^15^ Briefly, cells were incubated for 2 h in serum-free medium plus 50 µg/mL L-ascorbic acid, then labeled with 437.5 μCi/mL of 3.96 TBq/mmol L-[2,3,4,5-^3^H] proline for 16–18 h. Collagens were precipitated with ammonium sulfate, pepsinized, and run on 5% SDS-Urea-PAGE. To assess the deposition of collagen into the extracellular matrix, confluent osteoblasts were stimulated with DMEM containing 10% FBS, P/S, and 100 μg/mL ascorbic acid every other day for 14 days. 24 h prior to collection, cells were incubated with 260 μCi/mL of L-[2,3,4,5-^3^H]-proline in serum-free medium. Media was collected, and matrix collagens were serially extracted as described.^71^ Samples were loaded on 6% SDS-Urea-PAGE and balanced to give equal signal. The percentage of the radioactive signal in the media and combined matrix fractions was compared between genotypes (*n* = 3).

### Western blotting

Conditioned media were collected from near-confluent osteoblast or fibroblast cultures with protease inhibitors (P8340, Sigma-Aldrich) after 24 h incubation in serum-free medium. The conditioned media volume loaded was normalized to the cell counts. Extracellular matrix of differentiated osteoblasts was decellularized by lysing cells with extraction buffer (0.005% Triton X-100, 20 mmol/L NH_4_OH in 1× PBS) for thirty minutes at 37 °C. After washing three times with 1× PBS, the matrix was collected in T-PER lysis buffer (78510, Thermo Fisher, Waltham, MA) plus protease inhibitors. For bone lysates, epiphyses of femora and tibiae were removed, and marrow was flushed in PBS. Bones were pulverized in liquid nitrogen and extracted with T-PER buffer plus protease inhibitors. Samples were run on 4%–15% (media/matrix) or 4%–20% (bone lysates) TGX^TM^ precast gels (Bio-Rad, Hercules, CA) in LDS buffer (Thermo Fisher) and transferred to a 0.22 μmol/L nitrocellulose membrane. Blots were blocked in 5% BSA and incubated overnight with COL1A1 C-propeptide antibody (LF-42, a generous gift of Dr. Larry Fisher, NIH^72^) and washed, followed by 1 h incubation with 1:10 000 IRDye®-680RD goat anti-rabbit secondary antibody (926-68071, LI-COR, Lincoln, NE). Blots were imaged on an Odyssey Infrared imager CLx (LI-COR).

### Dermal and bone fibrils

Dermal biopsies from 2-month-old mice (heterozygotes) or 10-day-old pups (homozygotes) were fixed in 2.5% glutaraldehyde for 24 h, then transferred to phosphate buffer. Femurs were fixed in 4% paraformaldehyde (PFA, 50-980-487, Fisher Scientific) in Ca^2+^/Mg^2+^-free PBS for 2 days, then decalcified in 14% EDTA twice a week for 8 weeks before transferring to phosphate buffer. Fixed specimens were embedded and treated with 1% osmium tetroxide, then 2% uranyl acetate, followed by infiltration with Spurr’s plastic resin. Sections were examined by transmission electron microscopy (JFE Enterprises, Beltsville, MD). For fibril diameter measurements, 200 fibrils were measured from 3 to 4 separate images using digital calipers (Mitutoyo Corporation), then corrected for magnification.

### Sircol assay for collagen secretion

Calvarial or patient osteoblasts were grown to confluence, then incubated overnight in serum-free α-MEM + 1% P/S. After 24 h, conditioned media were collected with inhibitors (Sigma-Aldrich) and concentrated with spin columns (Amicon, Miami, FL, 10 K MWCO) to ≤200 µL. Secreted collagen was measured with the Sircol Collagen assay kit (Biocolor Carrickfergus, UK) using the manufacturer’s protocol, quantified at 540 nm on a NanoDrop 2000 (Thermo Fisher), and normalized to cell count.

### Collagen crosslinks

Trivalent (HP) and divalent (LP) crosslinks were measured in 2-, 6-, and 12-month femora (*n* = 3–6 each genotype) by mass spectrometry.^73^ Moles of total crosslinks were normalized by moles of collagen present.

### Micro-computed tomography (µCT) and 4-point bending

Femora of 2-, 6-, and 12-month-old mice were analyzed by µCT using a SkyScan1176 compact micro-CT scanner (MicroPhotonics, Allentown, PA) operating at 50 kV with an X-ray source current of 800 mA, as described previously.^73^ Femora were then biomechanically tested under four-point bending using a servohydraulic testing device (MTS 858; MiniBionix; MTS Systems Corporation, Eden Prairie, MN) as previously described.^73,74^ Further µCT analysis was performed on 6-month-old male hind limbs, pelvises of 3- and 4-month-old female mice, and homozygous HBM OI pups and their littermates.

### Bone mineralization density distribution (BMDD)

Femurs from 2-, 6-, and 12-month WT and HBM OI male mice were fixed in 70% ethanol and embedded undecalcified in polymethyl methacrylate (PMMA). The blocks were sawed, ground, polished, and carbon-coated for electron microscopy. BMDD measurements on cross-sectional area at the midshaft diaphysis were performed using quantitative backscattered electron imaging (qBEI) as previously described using a digital electron microscope (DSM 962, Zeiss, Oberkochen, Germany) equipped with a four-quadrant semiconductor backscattered electron detector.^19,75,76^ The BMDD parameters CaMean, CaPeak, CaWidth, CaLow, and CaHigh are as defined before.^19,77^ The same methods were used for the HBM OI patient bone sample, which was compared with historical controls. A section of the bone sample was stained with Goldner’s trichrome to visualize osteoid.

### In vitro mineral deposition assays

Primary calvarial or patient osteoblasts were grown in α-MEM supplemented with 10% FBS and 1% P/S until confluence. At confluence, cells were stimulated with osteoblast differentiation media three times a week for 28 days (mouse) or 6 weeks (human). Cells were washed in PBS and fixed in 4% PFA for 1 h. Calcium deposits were stained with 1% alizarin red in 2% ethanol for 30 min and washed extensively. Mineralization was quantified by solubilizing the stain with 5% SDS/0.5 N HCl for 10 min and measuring absorbance at 405 nm on a Victor X3 plate reader (Perkin Elmer, Waltham, MA).

### Static and dynamic histomorphometry

Mice were injected intraperitoneally with 30 mg/kg calcein (C0875, Sigma) at 10 days and 3 days prior to sacrifice. Femurs from 2-month male WT and HBM OI mice were fixed in 10% by neutral buffered formalin (HT5011, Sigma-Aldrich) for 48 h before washing with water and storing in 70% ethanol (*n* = 10, each genotype). The femurs were dehydrated in ascending grades of ethanol and embedded in glycol methacrylate. Five-micron sections were cut using a Leica RM2265 microtome equipped with a tungsten carbide steel blade. Sections were imaged using an Olympus BX 51 microscopy, equipped with a DP-73 digital camera. Unstained sections were used for calculation of dynamic histomorphometric parameters, while Goldner's Masson Trichrome stain or tartrate-resistant acid phosphatase (TRAP) stain with a fast green counter stain were used for static measures. Static and dynamic histomorphometry measurements were obtained from a region of interest 250 µm from the growth plate using OsteoMeasure software (OsteoMetrics, Decatur, GA) according to the standards established by ASBMR Histomorphometry Nomenclature Committee.^78^

### RNA collection and RNA-sequencing

RNA was collected at days 0, 10, 15, and 21 days of osteoblast differentiation from cultures derived from pooled newborn calvaria (*n* = 3 independent pooled cultures) or days 0, 7, 14, and 21 from human osteoblasts (*n* = 2). Osteoblast RNA was prepared using the Qiagen RNeasy kit (74106, Qiagen, Germantown, MD) with on-column DNase digestion. To isolate femoral RNA, femurs were removed as quickly as possible and immediately placed in RNAlater (AM720, Thermo Fisher). Femora were cleaned of soft tissue, and marrow was flushed with 1× PBS, then bones were crushed in liquid nitrogen. Total RNA from crushed bones was prepared using 1 mL TRI reagent (TR118, Molecular Research Center, Cincinnati, OH). An RNA integrity number of ≥7.0 was confirmed on an Agilent Bioanalyzer 2100 (Agilent, Santa Clara, CA).

For RNA-sequencing of mouse osteoblast samples, sample libraries were prepared using a TruSeq stranded mRNA kit (Illumina, San Diego, CA) at the NICHD Molecular Genomics Core. Detailed RNA-sequencing methods can be found in Supplementary Methods.

To confirm RNA-Seq results, RNA was reverse transcribed using a High-Capacity cDNA reverse transcription kit (4368813, Applied Biosystems, Waltham, MA). cDNA samples were run on the QuantStudio6 real-time PCR System (Applied Biosystems). Taqman probes used are listed in Table S1. Murine gene expression was normalized to three housekeeping genes (*Gapdh, B2m*, *Actb*); human gene expression was normalized to one housekeeping gene (*B2M*) due to low sample availability. Triplicate biological replicates were assayed in duplicate for each osteoblast differentiation prep; two to three osteoblast differentiation preps were tested for each gene.

### Osteocyte lacunae measurements

Osteocyte lacunae sections (OLS) were analyzed on 2-dimensional images of femora cross-sections from 2-, 6-, and 12-month WT and HBM OI male mice or of radial bone from human HBM OI obtained by qBEI as previously described.^79^ The five parameters characterized are defined as follows: (1) OLS density, the number of OLS per total bone area. (2) OLS-porosity, the total OLS area per total bone area. (3) OLS area: the mean value of the OLS areas per sample. (4) OLS-perimeter, the mean value of the OLS perimeters per sample. (5) OLS-aspect ratio (AR), the ratio of the long axis to the short axis of the OLS, used to measure OLS shape.

### Small-angle X-ray scattering (SAXS)

Longitudinal cuts of femurs from 2-month-old male mice, fixed and embedded undecalcified in PMMA, were measured using a Bruker Nanostar (Bruker Karlsruhe) for wide and small-angle X-ray scattering analyses, equipped with a 2-D Vantec-2000 detector and a microfocus X-ray source (IµS). This X-ray beam had a wavelength of 1.5418 Å (Cu Kα) and a focal spot size of 115 µm. SAXS data were collected at several positions of the cortex (5 positions in the lateral and medial cortex, each). The parameters T and ρ were determined as estimates of the mineral particle thickness and their degree of alignment and averaged over the five measurement points, respectively, to give a value in the lateral and medial cortex for each animal. Details of the evaluations are similar to previous work.^80,81^ Note that ρ varies between 0 and 1, whereby 0 means no alignment and 1 full alignment.

### Cell-matrix interactions

Calvarial osteoblasts were plated at 1 × 10^5^ cells per well of a 4-well chamber slide (154526, Lab-Tek II, Thermo Fisher). When confluent, cells were stimulated with osteoblast differentiation media for 21 days. After 21 days of differentiation, cells were removed by incubation with extraction buffer for 30 min at 37 °C. Matrices were washed extensively in PBS and stored at 4 °C in PBS if not used immediately. Osteoblasts were plated at 2.5 × 10^4^ cells per well and grown for 24 h before fixation in 4% PFA and staining.

### Immunofluorescence microscopy

Cells were plated in 4-well chamber slides and fixed with 4% PFA in PBS for 15 min. After washing in PBS, cells were permeabilized with 0.1% Triton X-100 and incubated with phalloidin-488 (A12379, Thermo Fisher) and vinculin (V9131, Sigma)/Alexa-555 antibodies (A21425, Thermo Fisher) for 30 min before extensively washing in PBS, staining with 1:5 000 DAPI (D9542, Sigma) and mounted with VectaShield Plus mounting medium (H-1900, Vector Laboratories, Newark, CA). Cells were imaged on an LSM 710 confocal microscope (Zeiss, Jena, Germany) (NICHD Microscopy and Imaging Core).

### Serum chemistry

Blood was collected from 2-month-old male mice by cardiac puncture, and serum was isolated by centrifugation at 1 500 × *g* for 15 min. Serum levels of type I procollagen N-propeptide (PINP) and tartrate-resistant acid phosphatase (TRAcP) were measured by Rat/Mouse PINP EIA kit (AC-33F1) or MouseTRAP^TM^ (TRAcP 5b) ELISA kit (SB-TR103, Immunodiagnostic Systems, East Boldon, UK), respectively, using the manufacturer’s protocol (*n* = 10, each genotype). HBM OI patient serum measurements were performed at the NIH Clinical Center.

### Statistical analysis

For qPCR analysis: Statistical analysis between genotypes was performed using a two-tailed Student’s *t*-test in Microsoft Excel. Statistical outliers were identified in GraphPad Prism v10.0.3 (GraphPad Software, Boston, Massachusetts, USA) using the ROUT method, *Q* = 1%. For qBEI analysis: Statistical analyses were carried out with GraphPad Prism v10.1.1 (GraphPad Software). Data were compared using unpaired *t*-tests or Mann-Whitney test as appropriate, and differences were considered statistically significant at *P* < 0.05. For SAXS analysis: Two-way ANOVA with repeated measures and Bonferroni *t*-test for pairwise comparisons was used to evaluate the effect of genotype and the difference between lateral and medial cortices.

## Supplementary information

Supplementary information

## Data Availability

Datasets produced in this study are available in the following databases: RNA-seq data: Gene Expression Omnibus GSE279055 (https://www.ncbi.nlm.nih.gov/geo/query/acc.cgi?acc=GSE279055), qBEI data (BMDD and OLS) produced in this study can be accessed at the LBG digital data repository for published research via creed.lbg.ac.at.
