# Supplementary material for: Murine model of high bone mass osteogenesis imperfecta exhibits bone matrix hyper-mineralization, misaligned mineral crystals, and altered osteoblast differentiation
Source: Bone Res. 2026 Jun 15;14:67. doi: 10.1038/s41413-026-00531-7 (PMC13269806; doi:10.1038/s41413-026-00531-7)
Supplement: Supplementary file 1 — Supplementary information [file 41413_2026_531_MOESM1_ESM.docx]

**Supplementary information**

**Procollagen C-propeptide Cleavage Impacts Bone Matrix Mineralization and Osteoblast Differentiation**

Aileen M. Barnes^1^, M. Helen Rajpar^1^, Joseph E. Perosky^2^, Stéphane Blouin^3,4^, Basma Khoury^2^, MaryAnn Weis^5^, Theresa Hefferan^6^, Alberta Derkyi^7^, Gali Guterman-Ram^1,8^, Ghazal Hedjazi^3^, Kiersten Campbell^9^, Chris Stephan^2^, David R. Eyre^4^, Ryan K. Dale^9^, Peter Fratzl^10^, Kenneth M. Kozloff^2^, Nadja Fratzl-Zelman^3,4^, Joan C. Marini^1^

**Supplementary information includes**:

Supplementary clinical information

Supplementary methods

Supplementary Figure S1

Supplementary Figure S2

Supplementary Figure S3

Supplementary Figure S4

Supplementary Figure S5

Supplementary Table S1

Supplementary Table S2

Supplementary Table S3

**Supplementary clinical information**

We previously described the first HBM OI proband, who has a *COL1A2* p.A1119T substitution (P2)^18^, when he was 12 years of age. He is currently 27 years old. On clinical exam at age 27 and over the years, he had normal facies with no dysmorphic features. His sclerae are light gray and he does not have dentinogenesis imperfecta. He has average stature with length that is 75^th^ percentile for an adult male and head circumference that is 98^th^ percentile for an 18-year-old.  His height is 181.2 cm, but his arm span of 191 cm, and upper and lower segment ratio of 1.04 both indicate mild shortening of his legs. He exhibited generalized hyperextensibility of extremities with the ability to place the palms of his hands flat on the ground while bending over at the waist.  Since he was initially reported, he has developed mild S-curve scoliosis, lumbar lordosis and thoracic kyphosis which are currently stable and has sustained numerous fractures secondary to an ATV accident, including a tripartite pelvic fracture and a lumbar vertebral fracture, as well as 8 fractured ribs with an associated pneumothorax. In addition to orthopedic procedures related to the ATV accident, he underwent a left humerus plating and 5^th^ finger pinning.

He received six cycles of bisphosphonate between ages 6-8 years but has not been on anti-resorptive medications since his mutation was identified. His L1 – L4 DXA (Hologic QDR 4500) average z-score at age 26 was z = +1.9 with a raw score average of 1.309 mg/cm. In contrast, he has had two L1 – L2 volumetric QCT (GE LightSpeed Ultra) analyses yielding

z-scores of -1.8 and -2.0, respectively, at ages 13 and 24 years. His spine bone density value was above 120 mg/cm^3^, indicative of normal bone density with minimal risk for osteoporotic fractures.

Serum calcium, magnesium, phosphate, alkaline phosphatase (ALP), bone-specific alkaline phosphatase (BSALP), osteocalcin and cholesterol were all in the normal range. Vitamin D levels have fluctuated over the years (between 16 and 54 ng/mL), mild to moderately low. He is currently taking vitamin D supplements. His audiological and cardiovascular (ECHO/EKG) exams are unremarkable.

On radiographs, his lower extremity showed evidence of mild osteopenia, minimal deformity of the right femur and right ischial tuberosity (**Figure 7A, left panel**) and growth arrest lines in fibula and tibia. Femur cortices remained thickened, without an osteopetrotic phenotype.  He has minimal scoliosis in the thoracic spine, slightly convex towards the left with slight exaggeration of the normal lumbar lordosis (**Figure 7A, middle panel**). He also has mild straightening of the normal thoracic kyphosis and a mild to moderate facet arthropathy in the lower lumbar spine (**Figure 7A, right panel**). Cervical CT skull showed no platybasia or basilar invagination.

**Supplementary Methods**

*Generation of HBM OI mice (expanded)*

The two amino acid residues constituting the *Col1a1* C-propeptide cleavage site were substituted into BAC RP24-338I21 (Children’s Hospital Oakland Research Institute, Oakland, CA) containing the entire mouse *Col1a1* genomic sequence.  The upstream arm of homology (5’ fragment) encompassed a 9.6 kb fragment from the *EagI* site in exon 10 to the *XhoI* site in exon 47. The downstream arm of homology (3’ fragment) encompassed a 3.5 kb *NdeI* fragment starting in intron 48 and ending downstream of the 3’UTR. A 500 bp PCR fragment was amplified from exon 47 with the *XhoI* site (uppercase) (F: tcCTCGAGgtcgcactggtgacagcg to intron 48, which contained the *NdeI* site (uppercase), and introduced an internal *BclI* site (underlined) (R:agtctacCATATGtgatcagaaagtggaatgtatgaaaacatgg). Site directed mutagenesis (SDM) using SDM-F: agatggtggccgctactaccggaccaatgatgctaacgtggttcgtga and SDM-R: tcacgaaccacgttagcatcattggtccggtagtagcggccaccatct (mutated base pairs are underlined), was performed according to the manufacturer’s instructions (QuikChange II XL Site-Directed Mutagenesis Kit (Agilent Technologies, Santa Clara, CA, USA)), and confirmed by direct sequencing. The three fragments were inserted into a pBlueScript II vector in which had been modified to contain the required restriction sites. A floxed Neo positive selection cassette was inserted into intron 48 using the *BclI* site introduced during the generation of the SDM fragment. A DTA (diphtheria toxin A-chain gene) negative selection element (pBJ101-DT^1^) was inserted after the 3’ fragment. The construct was linearized with *SacII* and electroporated into 129Sv/J cells. DNA was extracted from G418 resistant colonies and analyzed by PCR using a forward primer (F_a_) in the Neo cassette and a reverse primer (R_a_) in the sequence downstream of the 3’ fragment, to identify the clones with successful homologous integration of construct DNA.  Eighteen out of 144 clones were positive for the presence of the correctly sized 4 kb PCR product. Correct targeting was confirmed using Southern blotting. The DNA was digested with *BsrGI*, which generated a 4.5 kb fragment in the recombined allele and a 10 kb fragment in the wild-type (WT) allele, when hybridized with the external probe. Embryonic stem (ES) cells from clonal line #76 were checked for aneuploidy using standard karyotype analysis and found to be normal. C57Bl/6J blastocysts were microinjected with ES cells from line #76 and #3, resulting in 1 male and 2 female chimeras from #76 and 4 male chimeras from #3.

*Staining sections for static and dynamic histomorphometry*

Unstained sections were used for measurement of calcein label for calculation of dynamic histomorphometric parameters, including mineral apposition rate, bone formation rate, adjusted apposition rate, and mineralization lag time. Sections stained with a modified Goldners Masson Trichrome stain were used for measurement of static histomorphometric parameters including bone volume; cortical width; osteoid surface, thickness, and volume; eroded surface; osteoblast number and surface; and trabecular number, thickness, and separation. Sections stained with tartrate resistant acid phosphatase (TRAP) stain with a fast green counter stain were used for measurement of osteoclast number and surface.

*RNA Sequencing methods (expanded)*

For RNA sequencing of mouse osteoblast samples, sample libraries were prepared using a TruSeq stranded mRNA kit (Illumina, San Diego, CA) at the NICHD Molecular Genomics Core. Thirty to fifty million 100-bp paired-end reads were sequenced for each mouse sample. Sequencing quality was inspected with FastQC v0.11.9 and MultiQC v1.10.1 with no issues identified. Adapters were trimmed, and light quality trimming was performed, using cutadapt v3.4^2^ with additional parameters -a AGATCGGAAGAGCACACGTCTGAACTCCAGTCA -A AGATCGGAAGAGCGTCGTGTAGGGAAAGAGTGT -q 20 --minimum-length 25. Reads were aligned to the GENCODE vM18 assembly (i.e., mm10) with STAR v2.7.8a^3^ with the following additional parameters as recommended by the ENCODE consortium: --outFilterType=BySJout --outFilterMultimapNmax=20 --alignSJoverhangMin=8 --alignSJDBoverhangMin=1 --outFilterMismatchNmax=999 --outFilterMismatchNoverReadLmax=0.04   --alignIntronMin=20 --alignIntronMax=1000000 --alignMatesGapMax=1000000 --outSAMunmapped=None. Aligned reads were counted in genes with featureCounts (subread package, v2.0.1^4^) using the GENCODE vM18 GTF annotations and additional parameter -s2. Raw counts were loaded into DESeq2 v1.30.1^5^ running under R v4.0.4. Variance was estimated on each day separately, including a term for batch (that is, using the model ~genotype + batch) and extracting results just for the genotype contrast (HBM vs WT). Statistically significant (Benjamini-Hochberg adjusted p-values < 0.1) upregulated and downregulated genes at each timepoint were assessed for overrepresentation of gene ontology Biological Process (BP) terms using the clusterProfiler v3.18.1 R package^6^. Dotted version names (i.e., a “.1” suffix) on GENCODE gene IDs were removed to match the mouse OrgDb Ensembl IDs.

**References**

1 McCarrick, J. W., 3rd, Parnes, J. R., Seong, R. H., Solter, D. & Knowles, B. B. Positive-negative selection gene targeting with the diphtheria toxin A-chain gene in mouse embryonic stem cells. *Transgenic research* **2**, 183-190 (1993).

2 Martin, M. Cutadapt removes adapter sequences from high-throughput sequencing reads. *2011* **17**, 3 (2011). <https://doi.org/10.14806/ej.17.1.200>

3 Dobin, A. *et al.* STAR: ultrafast universal RNA-seq aligner. *Bioinformatics* **29**, 15-21 (2013). <https://doi.org/10.1093/bioinformatics/bts635>

4 Liao, Y., Smyth, G. K. & Shi, W. featureCounts: an efficient general purpose program for assigning sequence reads to genomic features. *Bioinformatics* **30**, 923-930 (2014). <https://doi.org/10.1093/bioinformatics/btt656>

5 Love, M. I., Huber, W. & Anders, S. Moderated estimation of fold change and dispersion for RNA-seq data with DESeq2. *Genome Biol* **15**, 550 (2014). <https://doi.org/10.1186/s13059-014-0550-8>

6 Yu, G., Wang, L. G., Han, Y. & He, Q. Y. clusterProfiler: an R package for comparing biological themes among gene clusters. *OMICS* **16**, 284-287 (2012). <https://doi.org/10.1089/omi.2011.0118>


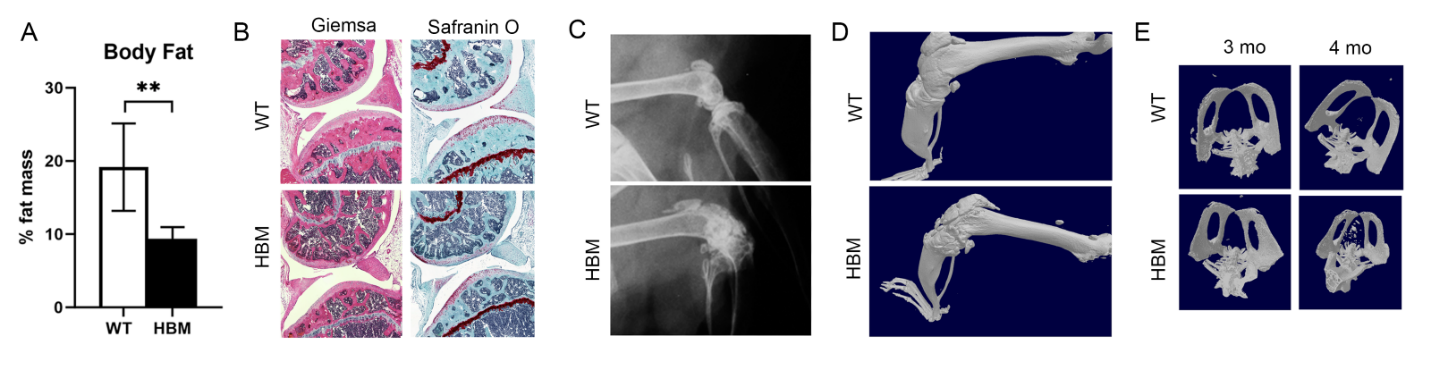


**Supplementary Figure S1: HBM OI mice have increased soft tissue calcifications**

A) The total fat mass percentage, as measured by DXA, remained significantly decreased in 2-year old HBM OI mice.  (WT = white bars, HBM OI = black bars) B) Histology of the knee joint of 6-month old mice by hematoxylin and eosin staining (left panels) and safranin O/light green staining (right panels), shows no signs of osteoarthritis in either genotype.  C) Radiographs of 12-month old mice show calcification at the knee joint.  D)  mCT 3-dimensional rendering of 6-month old hind limbs confirm calcification of the patellar tendon.  E) mCT 3-dimensional rendering of pelvises from breeding-age female mice displays a narrower birth canal and excess calcifications.


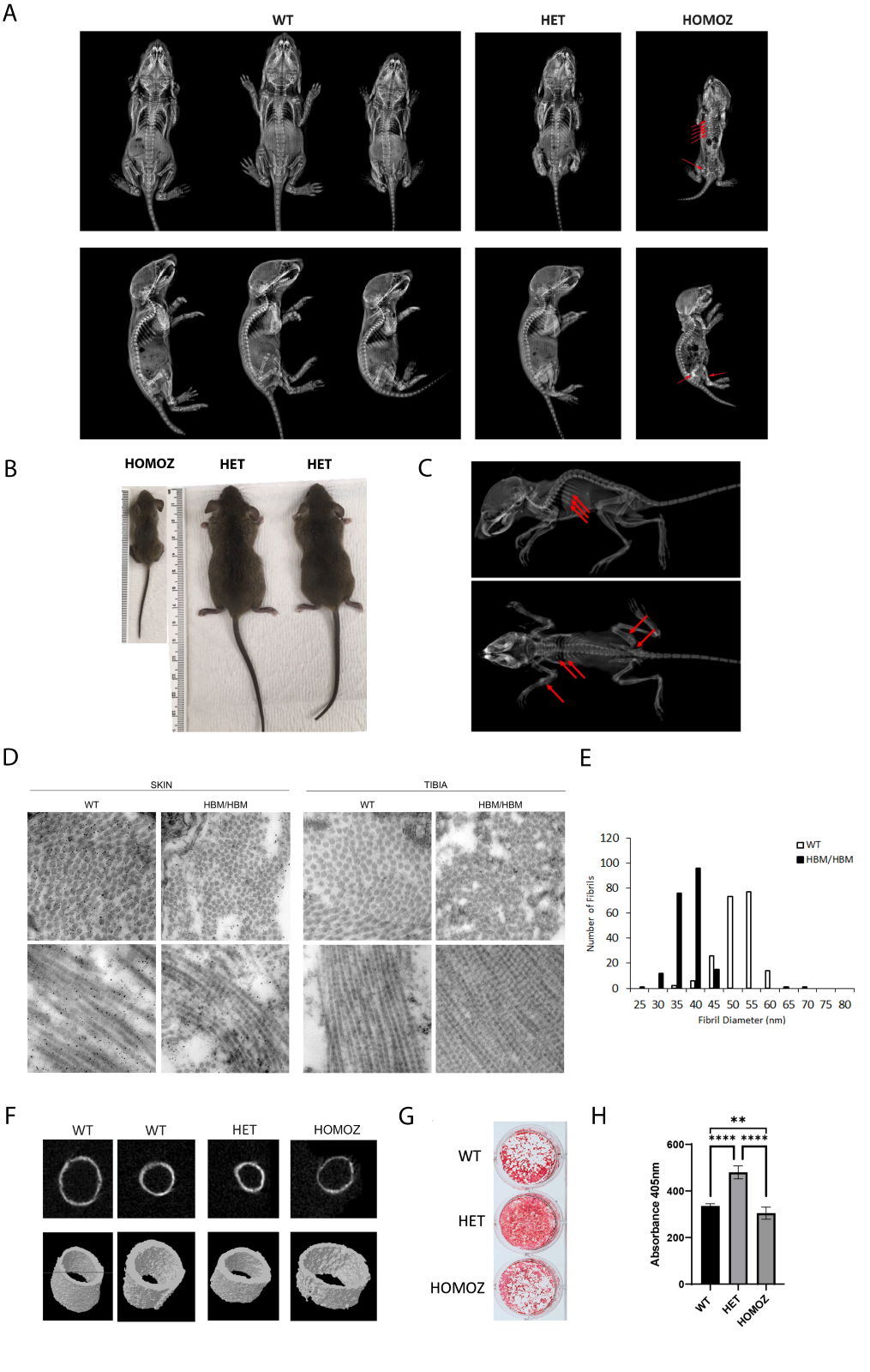


**Supplementary Figure S2: HBM OI/HBM OI homozygous mice have increased fractures and smaller size** A) Radiographs of a 10-day old homozygous HBM OI/HBM OI pup and its littermates, exhibit multiple fractures (red arrows) and greatly decreased size.  B)  Size comparison of 5-week-old male homozygous pup and its heterozygous HBM OI littermates.  C) Radiographs of 5-week-old homozygous pup shows multiple rib fractures (top panel, red arrows) and rib, radius/ulna, femur and pelvic fractures (bottom panel, red arrows).   D) Electron micrographs of skin and bone collagen fibrils from the 10-day-old homozygous HBM OI/HBM OI pup and its WT littermate.  Fibril cross-sectional diameters are noticeably smaller in HBM OI/HBM OI.  E) Histogram of WT and HBM OI/HBM OI dermal fibril measurements (n=200) reveals a significant decrease in fibril diameter in the homozygous mouse.  F) mCT 3-dimensional rendering of the femur diaphysis of the 10-day-old HBM OI/HBM OI pup and its littermates, displays a decreased bone surface in both heterozygous and homozygous HBM OI mice.   G) Alizarin red staining of pooled newborn calvarial osteoblasts differentiated *in vitro* for 28 days in culture displays increased mineral staining in HBM OI mice but decreased staining in HBM OI/HBM OI mice.  H) Quantitation of alizarin red staining of newborn calvarial osteoblasts. (*p<0.05, **p<0.01, ***p <0.005, ****p<0.001)


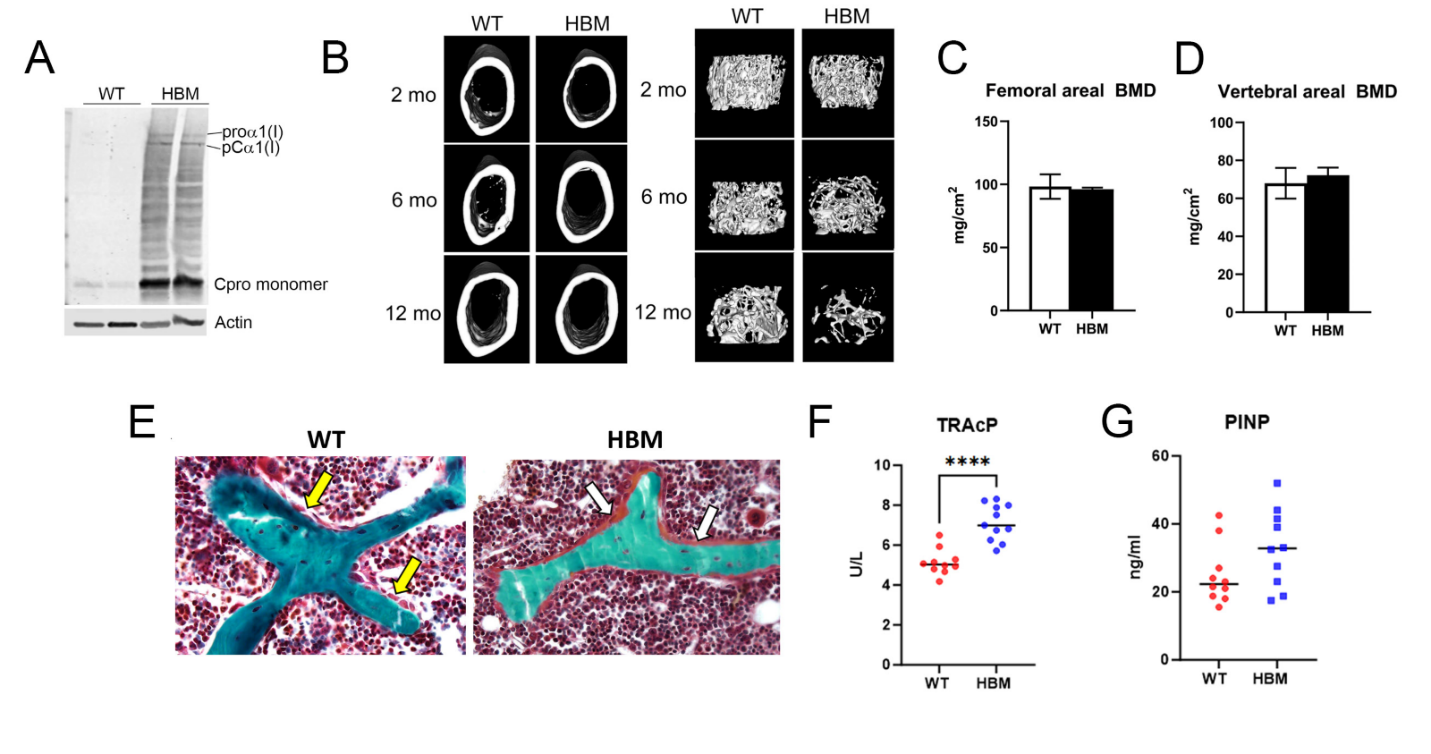
**Supplementary Figure S3: Older HBM OI mice still retain the C-propeptide in long bone and have a normal areal bone density**

A) COL1A1 C-propeptide western blot of long bone tissue (femora and tibiae) lysate from 1-year old mice, revealing that pC-α1(I) and monomeric C-propeptide remain in bone tissue over time.  B) 3-Dimensional µCT reconstructions show slightly thinner cortex and a large decrease in trabeculae over time in HBM OI bone.   C) Femoral aBMD is normal in HBM OI vs WT at 2-years of age. D) Vertebral aBMD is normal in 2-year old HBM OI mice.  E) Images of Goldner trichrome stained trabeculae show mature osteoblasts lining the bone surface in WT (yellow arrows) and thick osteoid seams in HBM OI (white arrows). F) TRAcP levels in serum are significantly increased in HBM OI mice. G) PINP serum levels show no significant difference between genotypes.  (**p<0.01, ****p<0.001)


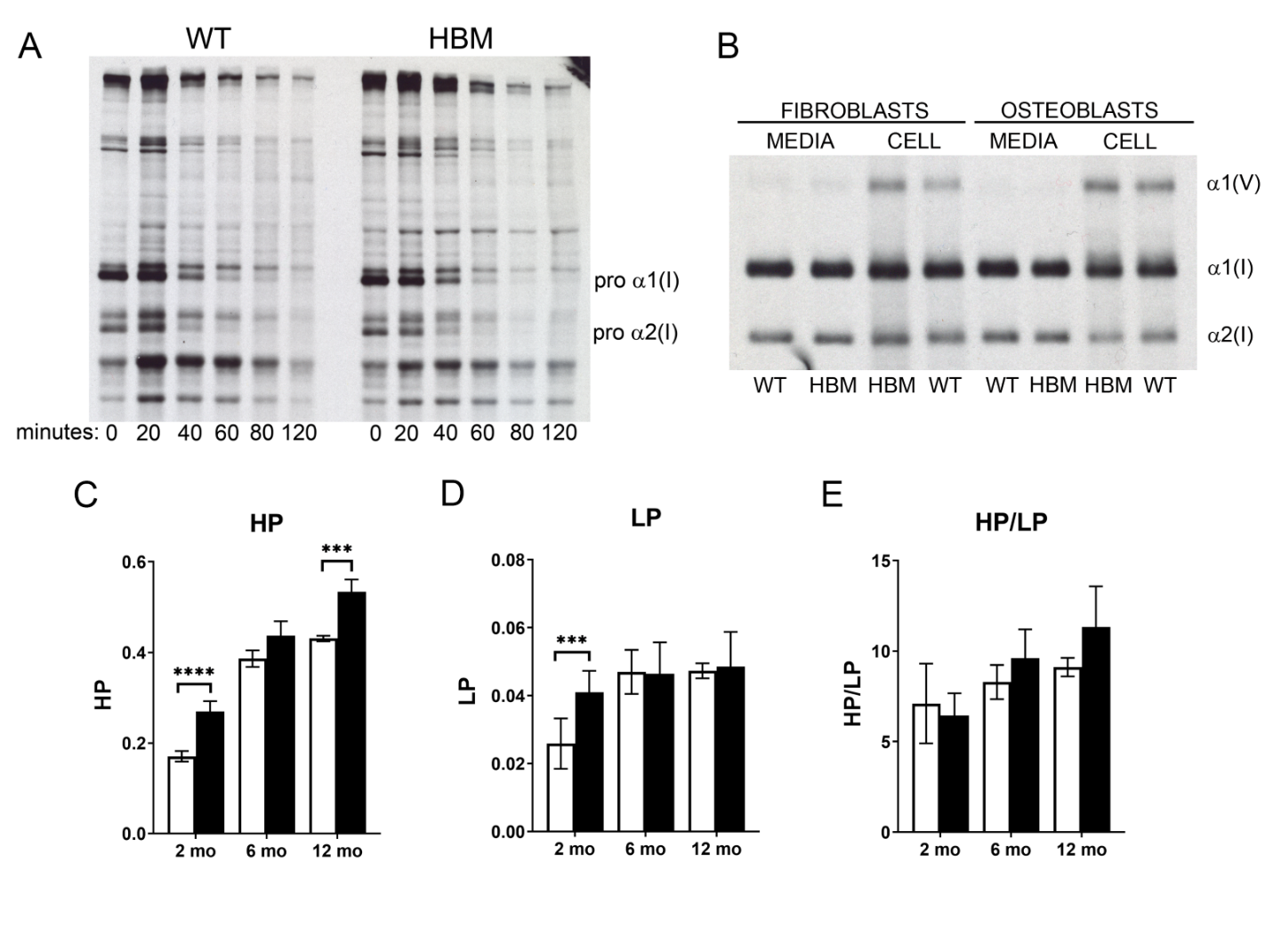


**Supplementary Figure S4: Type I collagen in HBM OI is normally processed yet has altered crosslinking**.

A) Procollagen from HBM OI osteoblasts is associated normally into trimers, with pro alpha chains secreted by 60 minutes in both genotypes, as shown by pulse-chase assay. B) Steady-state biochemistry of pepsin-digested secreted and intracellular type I collagen chains shows normal migration in HBM OI fibroblasts and osteoblasts. C) Trivalent HP crosslinks are significantly increased at 2- and 12-months of age.  D) Divalent LP crosslinks are reduced at 2-months but normalize by 6-months.  E) The HP/LP ratio is unaffected in HBM OI mice. (WT = white bars, HBM OI = black bars) (*p<0.05, **p<0.01, ***p <0.005, ****p<0.001)


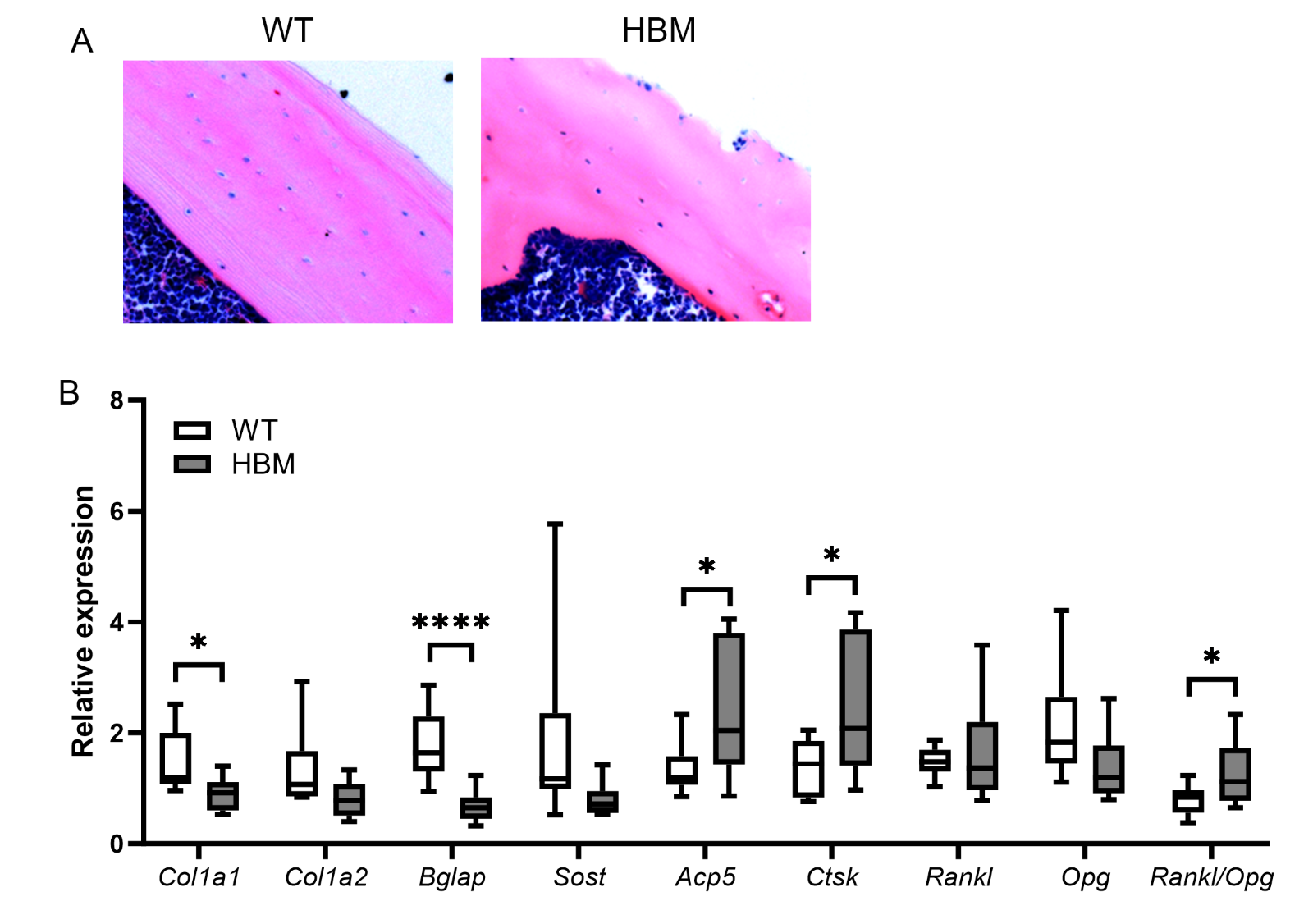


**Supplementary Figure S5: HBM OI bone has fewer osteocytes and altered osteocyte gene expression**

A) Hematoxylin and eosin-stained femur sections from 2-month mice show fewer osteocytes in HBM OI cortical bone.  B) Osteoblastic gene transcripts *Col1a1*, *Col1a2*, *Bglap*, and *Sost* are down-regulated in RNA from murine HBM OI bone, while transcripts promoting osteoclastogenesis *Acp5*, *Ctsk*, and *Rankl/Tnfsf11* are all increased. Despite a slight decrease in *Opg/Tnfrsf11b* expression, the *Rankl/Opg* ratio is significantly increased, suggesting promotion of osteoclastogenesis (n=9-11 mice per genotype). (WT = white bars, HBM OI = dark grey bars) (*p<0.05, ****p<0.001)


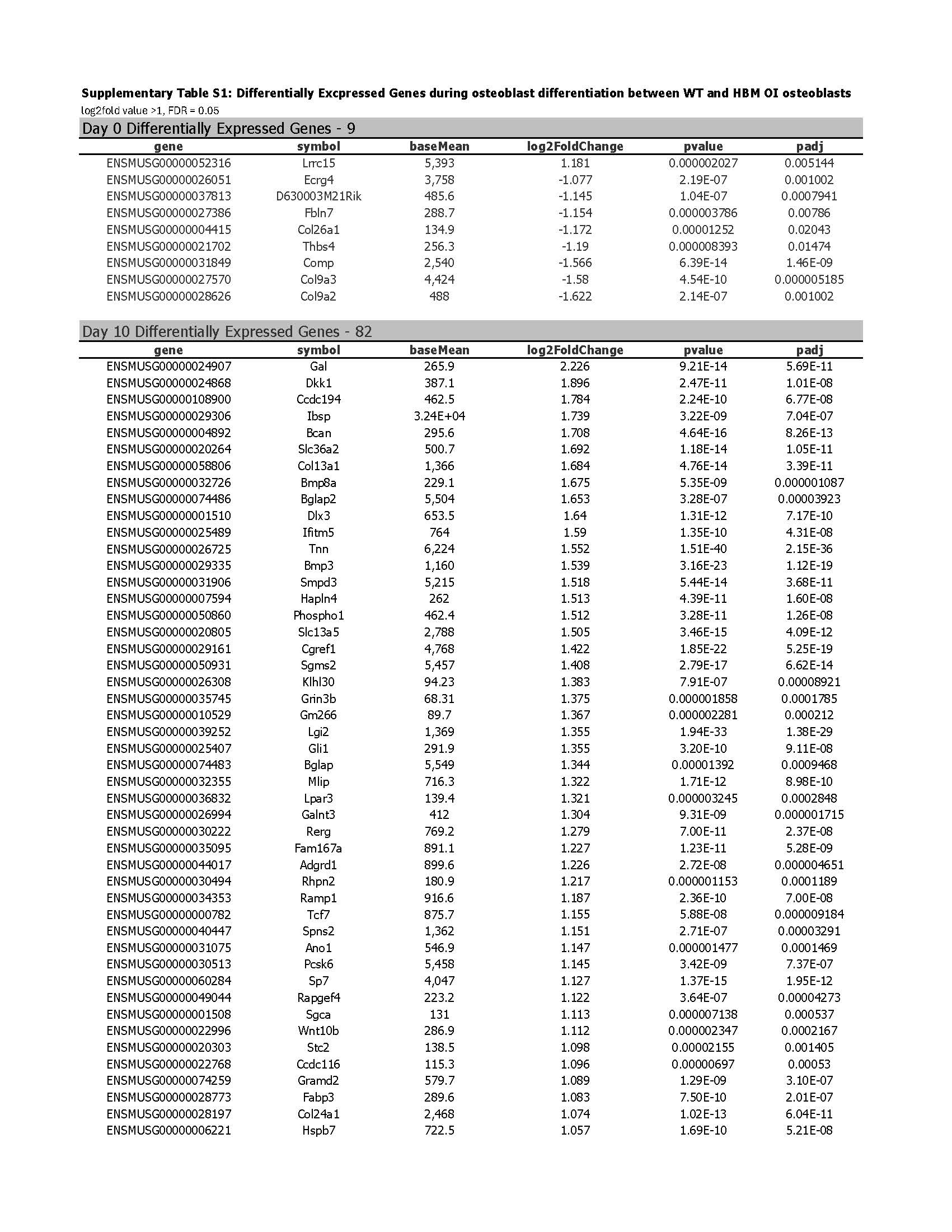


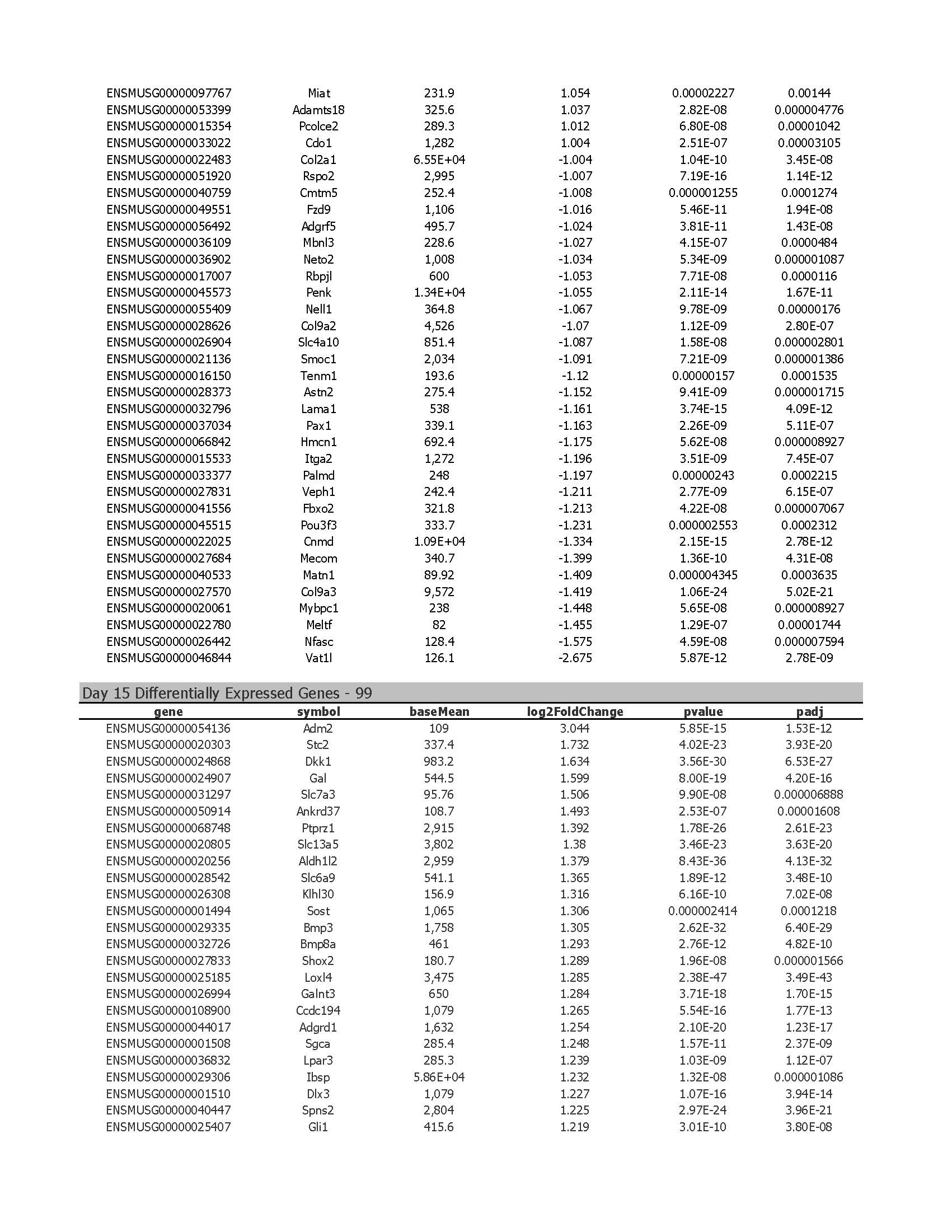

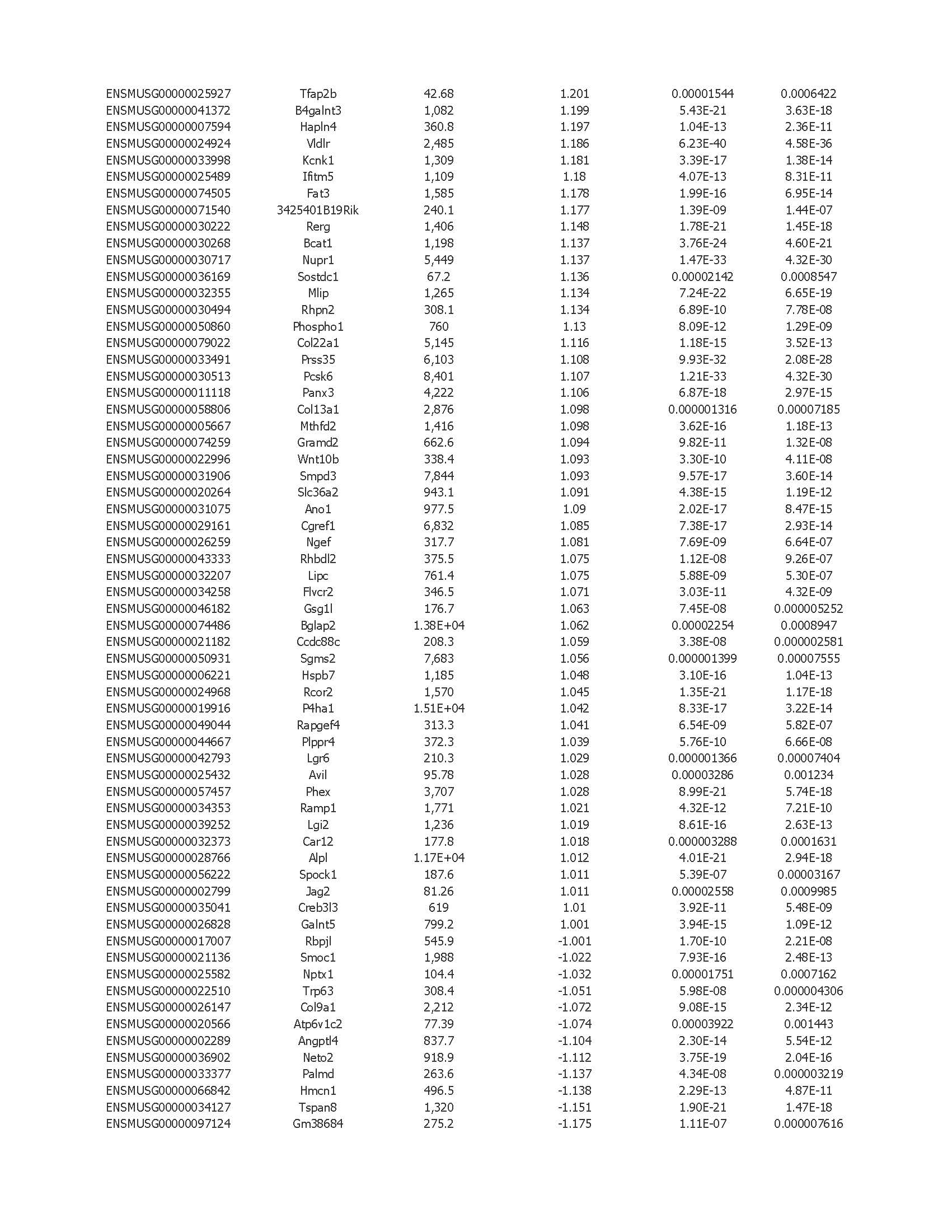

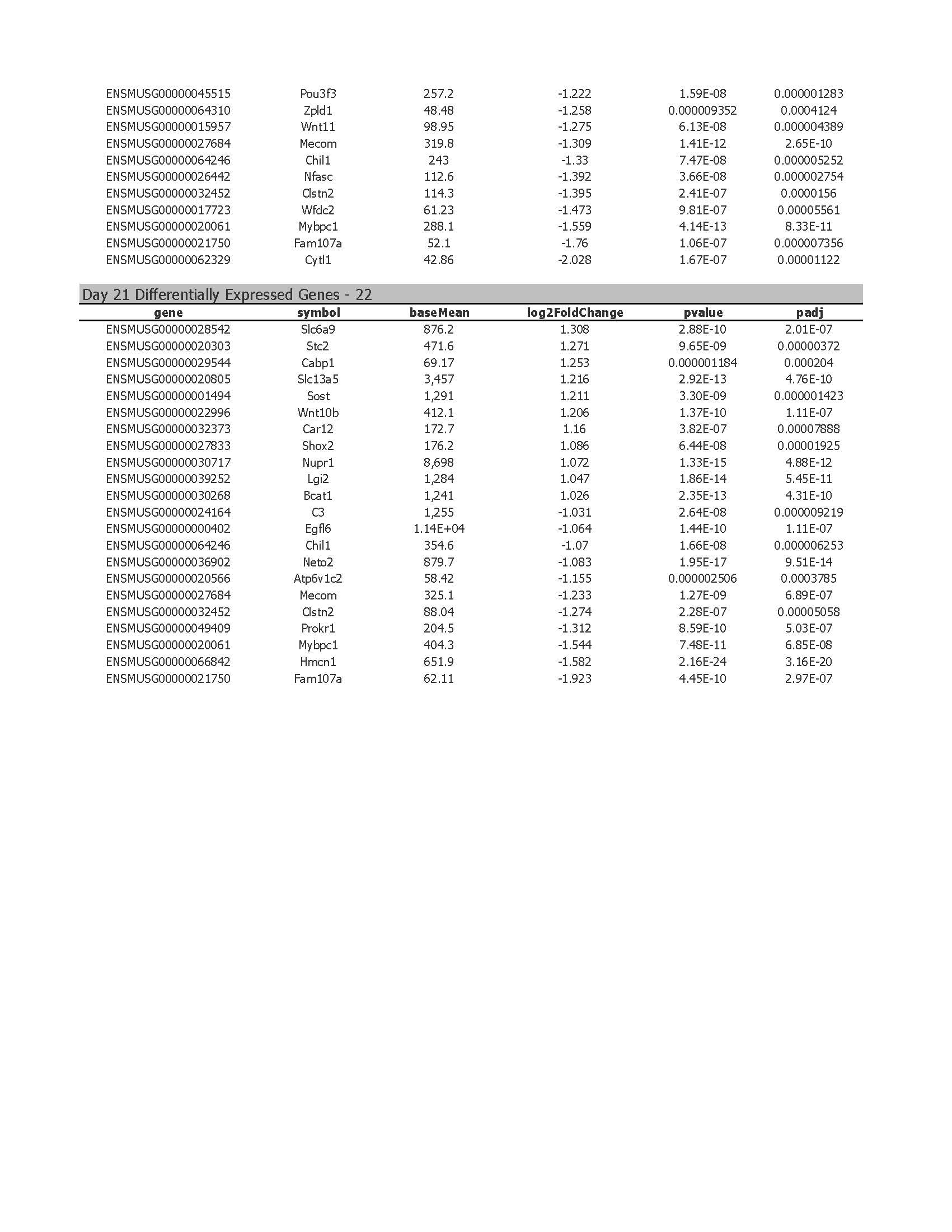


**Supplementary Table S2:** **Osteocyte Lacunae Section (OLS) Parameters**

|  | **Age WT mice (months)** | | |  | **Age HBM-OI mice (months)** | | |  | **Two-way ANOVA**  **(p-value)** | | |
| --- | --- | --- | --- | --- | --- | --- | --- | --- | --- | --- | --- |
|  | **2** | **6** | **12** |  | **2** | **6** | **12** |  | **Inter-action** | **Age** | **Genotype** |
| Mean  Porosity | 1.59  (0.21) | 1.03  (0.11) | 0.87  (0.07) |  | 1.26  (0.22) | 0.89  (0.12) | 0.79  (0.15) |  | **0.02*** | **< 0.0001** | **<0.0001** |
| Mean  Density | 1093  (67.11) | 502.5  (53.13) | 432.2  (45.90) |  | 714.7  (84.29) | 378.8  (34.53) | 352.7  (59.70) |  | **< 0.0001** | **< 0.0001** | **< 0.0001** |
| Mean  OLS area | 14.78  (1.40) | 14.51  (1.08) | 13.22  (0.55) |  | 17.79  (1.98) | 16.78  (1.48) | 14.74  (1.24) |  | 0.21 (ns) | **< 0.0001** | **< 0.0001** |
| Mean  OLS perimeter | 15.73  (0.84) | 15.39  (0.81) | 14.82  (0.56) |  | 17.44  (1.06) | 15.97  (0.54) | 15.49  (0.67) |  | **0.03*** | **< 0.0001** | **< 0.0001** |
| Mean  OLS AR | 2.61  (0.15) | 3.01  (0.26) | 3.15  (0.27) |  | 2.76  (0.14) | 2.94  (0.19) | 3.21  (0.12) |  | 0.184 (ns) | **< 0.0001** | 0.34 (ns) |

Values are given in Mean ± (SD)

**Supplementary Table S3: Human HBM OI Bone parameters**

| **BMDD parameters** | ***COL1A2* p.A1119T (radius)** | | **Transiliac bone –**  **Cortical references (n=25)(Hartmann *et al*, 2021)** |  |  |
| --- | --- | --- | --- | --- | --- |
| CaMean  [wt% Ca] | 24.74 | | 22.96 ± 0.57 |  |  |
| CaPeak  [wt% Ca] | 26.69 | | 23.89 ± 0.56 |  |  |
| CaWidth  [Δwt % Ca] | 3.81 | | 4.22 ± 0.41 |  |  |
| CaLow  [% bone area] | 7.69 | | 5.33 ± 1.33 |  |  |
| CaHigh  [% bone area] | 38.48 | | 4.71 ± 2.78 |  |  |
| **OLS**  **parameters** | ***COL1A2* p.A1119T**  **(25.5 years old)** | | **Reference Cortical bone(Blouin *et al*, 2023)** | **Z-score** |  |
| OLS-Porosity (%) | | 1.04 | 0.50 ± 0.14 | +3.7 |  |
| OLS-Density (number/mm^2^) | | 267 | 220 ± 58 | +0.8 |  |
| OLS-Area (µm^2^) | | 33.37 | 19.18 ± 3.27 | +4.3 |  |
| OLS-Perimeter (µm) | | 25.31 | 19.25 ± 2.12 | +2.9 |  |
| OLS-Aspect ratio | | 1.98 | 2.56 ± 0.29 | -2 |  |
